# Tracking water dimers in ambient nanocapsules by vibrational spectroscopy

**DOI:** 10.1073/pnas.2212497119

**Published:** 2022-12-01

**Authors:** Alexander Y. Hwang, Rohit Chikkaraddy, David-Benjamin Grys, Oren A. Scherman, Jeremy J. Baumberg, Bart de Nijs

**Affiliations:** ^a^Department of Physics, NanoPhotonics Centre, Cavendish Laboratory, University of Cambridge, Cambridge CB3 0HE, United Kingdom; ^b^Department of Chemistry, Melville Laboratory for Polymer Synthesis, University of Cambridge, Cambridge CB2 1EW, United Kingdom

**Keywords:** water clusters, water dimer, vibrational spectroscopy, cucurbituril

## Abstract

Nanoconfined few-molecule water clusters are invaluable systems to study fundamental aspects of hydrogen bonding. Unfortunately, most experiments on water clusters must be performed at cryogenic temperatures. Probing water clusters in noncryogenic systems is however crucial to understand the behavior of confined water in atmospheric or biological settings, but such systems usually require either complex synthesis and/or introduce many confounding external bonds to the clusters. Here, we show that combining Raman spectroscopy with the molecular nanocapsule cucurbituril is a powerful technique to sequester and analyze water clusters in ambient conditions. We observe sharp peaks in vibrational spectra arising from a single rigid confined water dimer. The high resolution and rich information in these vibrational spectra allow us to track specific isotopic exchanges inside the water dimer, verified with density-functional theory and kinetic population modeling. We showcase the versatility of such molecular nanocapsules by tracking water cluster vibrations through systematic changes in confinement size, in temperatures up to 120° C, and in their chemical environment.

## Significance

Modeling water’s dynamic bond network and accurately predicting physical properties is challenging. As a result, small water clusters are crucial templates to understand hydrogen bonding at a simpler level. Unfortunately, such clusters can only be studied under cryogenic conditions and often with uncontrolled charge or size. Here, we introduce a technique to trap and analyze neutral water clusters in ambient conditions, by encapsulating them in barrel-shaped molecules (cucurbiturils) and measuring them with Raman spectroscopy. We observe a confined water dimer and demonstrate facile tuning of its environment and even isotopic composition, paving the way for probing ambient water cluster dynamics in situations difficult to access in previous experiments.

Despite water’s significance in nearly all chemical and biological processes, many open questions remain about its unique hydrogen bonding network. In ambient bulk water, rapid fluctuations and many degrees of freedom hinder the systematic characterization of interactions. Confined water, with greatly reduced degrees of freedom, is hence a powerful tool to study hydrogen bonding of ambient water in more detail. Confined water is ubiquitous in nature and has broad applications in areas such as biochemistry, chemistry, surface science, climate science, novel materials, and geology. The most extreme confinement produces *few-molecule water clusters*, which are invaluable systems to probe the fundamentals of hydrogen bonding (1–3).

While much work exists on hydrogen bonding in water clusters, most experimental methods require cryogenic experiments with either matrix isolation (4, 5), molecular beams (2, 6–11), or nanodroplets (12, 13). Important systems in biochemical or atmospheric environments however involve clustered water under ambient conditions. Theoretical studies predict rich dynamics when water clusters move from low temperatures to ambient conditions, including ‘melting’ of solid-like clusters to liquid-like phases (14). Experimental studies of ambient clusters are rare, and full temperature-dependent measurements of neutral water clusters have not yet been conducted (14). Room temperature water cluster systems exist and provide useful physical insights (15) but usually involve several extra bonds between water clusters and their environments. While fullerene systems are a notable exception, they encapsulate water monomers, dimers, and trimers with nontrivial chemistry (16). Beyond the requirement of cryogenic temperatures, current methods to study water clusters have significant other experimental limitations. For example, with molecular beams, it can be difficult to measure neutral (6, 17) (nonionized) water clusters and also size-select (2, 7, 9) for a desired water cluster, though recent works (8, 17) aim to address these challenges.

**Fig. 1.**
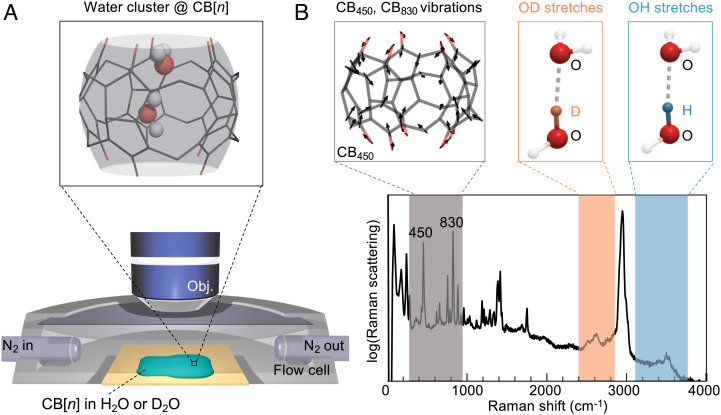
Raman spectroscopy of CB[*n*] during drying. (*A*) Schematic experiment: Raman spectra are recorded from a CB[*n*] solution in either H_2_O or D_2_O whilst it dries inside a nitrogen-flow cell. (*B*) Typical Raman spectrum highlighting the three vibrational regions of interest (shaded). The large peak between OD and OH stretch bands at 2,900 cm^−1^ originates from C–H stretch vibrations.

Here, cucurbituril (CB[*n*]) (Fig. 1*A*), a pumpkin-shaped macrocyclic inclusion complex formed from *n* glycouril units, is used to isolate and analyze water clusters in ambient conditions. Like most supramolecular structures, CB[*n*] naturally hosts water clusters inside its cavity (‘water@CB[*n*]’) for thermodynamic stability (18–20). While the water cluster molecules near CB[*n*] electronegative portals are anchored to carbonyls through hydrogen bonds, the remaining portions of the cluster are encapsulated inside the largely electron-deficient cavity interior without additional confounding bonds (18, 19, 21). Earlier experimental studies of these confined water clusters focused on the thermodynamics of confined water and their contribution to CB[*n*] host–guest binding processes (22–25). Meanwhile, research focussed directly on the details of hydrogen bonding within water@CB[*n*] has been limited to theory (26–28).

Raman spectroscopy is a powerful tool for probing hydrogen bonding in water (29). Gas-phase OH-stretch vibrations are observed at ∼3,700 cm^−1^ (OD at ∼2,700 cm^−1^) but can downshift by as much as 600 cm^−1^ upon hydrogen bonding, when electron densities are pulled away from the OH (OD) bond (2). Here, we use this technique to systematically study the behavior of discrete water clusters trapped in molecular cages of CB[*n*]. The smallest CB homologue CB[5] is of particular interest as its small cavity can only accommodate two water molecules (*SI Appendix*, Table S1), allowing us to probe an isolated hydrogen bond in ambient conditions. We connect our findings to density functional theory (DFT) and rate equation models that track H–D isotope exchange in these dimers. Finally, we highlight the versatility of this system and how it enables new experiments on ambient water clusters, demonstrating control of the degree of confinement, the effects of temperature, and how bonding interactions of such confined water clusters can be modified.

## Results and Discussion

### Raman Spectra of CB[5]-Confined Water Dimers.

As CB[5] in H_2_O or D_2_O dries (Fig. 1*A*), changes in Raman spectra are observed in three distinct regions (Fig. 1*B*): CB vibrations at ∼450 cm^−1^ (CB_450_, ring scissor) and 830 cm^−1^ (CB_830_, ring deformation) (30) (gray shaded), OD stretches from 2,300 to 2,800 cm^−1^ (orange shaded) (31), and OH stretches from 3,100 to 3,800 cm^−1^ (blue shaded) (31). The CB_450_ and CB_830_ peak frequencies are sensitive to the CB[*n*] guest contents (32), while OD and OH stretches shift to lower frequencies with stronger hydrogen bonds.

**Fig. 2.**
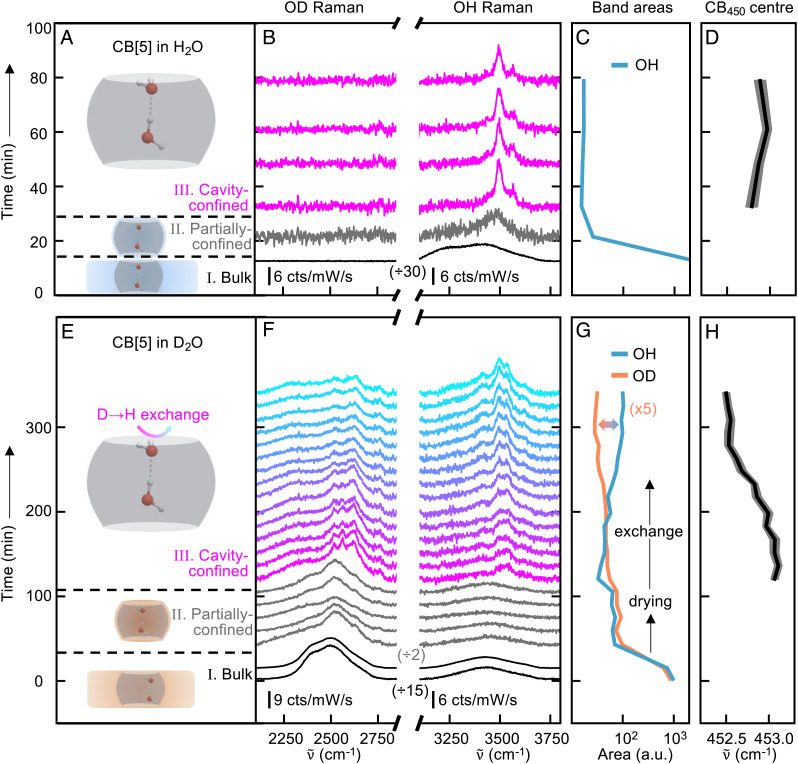
Cavity-confined water signals in CB[5], for (*Top*) H_2_O and (*Bottom*) D_2_O. (*A*, *E*) When CB[5] dries from H_2_O or D_2_O, three distinct stages are observed, as labeled. (*B*, *F*) In stage I, bulk water is observed (black curves). Stage II shows partially confined water (gray curves). Finally, stage III exhibits narrow bands, indicating cavity-confined water (magenta-cyan curves). (*C*, *G*) Integrated areas of the OH and OD bands showing for H_2_O an initial drop in phases I,II and a stable signal in III (*C*), but for D_2_O isotopic exchange is observed in phase III (*G*). (*D*, *H*) CB_450_ peak position remains stable over time for H_2_O in phase III (*D*), but shifts in response to D→H isotope exchange (*H*).

The time-dependent Raman spectra reveal three stages in the CB[5] drying process (Fig. 2 *A* and *E*). In Stage I, bulk water is present, resulting in broad OH and OD stretch peaks (Fig. 2 *B* and *F*, black curves). The D_2_O experiment shows broad OD peaks of bulk D_2_O and comparatively smaller OH peaks from HDO (31), while the H_2_O experiment shows the characteristic peaks of bulk H_2_O. Next, in a ‘partially confined water’ step (stage II), the integrated signals of both bands strongly decrease as the bulk water evaporates (Fig. 2 *C* and *G*). The OH and OD bands (Fig. 2 *B* and *F*, gray curves) still exhibit broad bands but shift to a higher-frequencies characteristic of confined but not clustered water (33, 34). Finally, the dry nitrogen gas flow removes the remaining free water in the voids, evidenced by further reductions in the integrated peaks (Fig. 2 *C* and *G*), leaving only ‘cavity-confined water’ or ‘water@CB[5]’ behind (stage III). These latter peaks have a distinctly narrower FWHM (from ∼200 cm^−1^ to <20–50 cm^−1^, *SI Appendix*, Tables S2 and S3) characteristic of discrete water clusters (2, 35). The small peak near 2,330 cm^−1^ comes from free N_2_ in the air within the focal volume of the laser.

In stage III, the H_2_O@CB[5] signals remain stable in time and no changes are observed in the CB[5] Raman lines (Fig. 2*D*). In contrast, for D_2_O experiments, further dynamics are visible, with Raman bands showing evidence of D→H isotope exchange over time; the water cluster signal dominated by narrow OD peaks (Fig. 2*F*, magenta curves) is replaced by narrow OH peaks (Fig. 2*F*, cyan curves), as seen in integrated peak areas vs. time (Fig. 2*G*). Concurrently, a 0.5 cm^−1^ downshift of the CB_450_ vibration is observed (Fig. 2*H*), indicating that the isotopic exchange occurs in close proximity to CB[5]. Removing samples from the low-humidity environment results in the sharp OH peaks reverting back over hour timescales to the broad OH features observed for stage II (*SI Appendix*, Fig. S1). These broad bands are similar to those observed in previous reports on water complexation with CB[*n*] (26, 36), showing that the dry nitrogen flow is vital in counteracting the hygroscopic properties of CB[*n*] solids. Reintroducing the dry nitrogen rapidly reverts the sample back to stage III, demonstrating that stage II water is not tightly bound and therefore unlikely to reside within the CB[*n*] cavities.

Previous experiments and theory agree that different sizes of CB[*n*] trap different numbers of water molecules (*SI Appendix*, Table S1) with CB[5] expected to only incorporate two water molecules. This means that stage III water@CB[5] signals likely arise from discrete water dimers: (H_2_O)_2_ or isotopologues thereof. We propose (below) that this sequestered water dimer is in a rigid state as a result of the highly restrictive space in the CB[5] cavity (4.4 Å equatorial diameter) (20) compared to the size of a water molecule, which is why the Raman peaks are narrow. Larger CB[*n*] homologues accommodate more water molecules (3–13) with milder spatial restriction, allowing water molecules and hydrogen bonds to fluctuate, more closely resembling the behavior of bulk water (20, 22). Because of this, broader peak distributions are expected for the larger CB[*n *= 6–8] homologues from inhomogeneous broadening, as confirmed experimentally below. While recent works demonstrate trapping of water dimers in supramolecular structures (37, 38), the result here for (H_2_O)_2_@CB[5] is the first to analyze the vibrational spectra of the confined dimer in depth. We note that few works discuss Raman spectra of water clusters rather than infrared spectra (7).

### Theoretical Model.

**Fig. 3.**
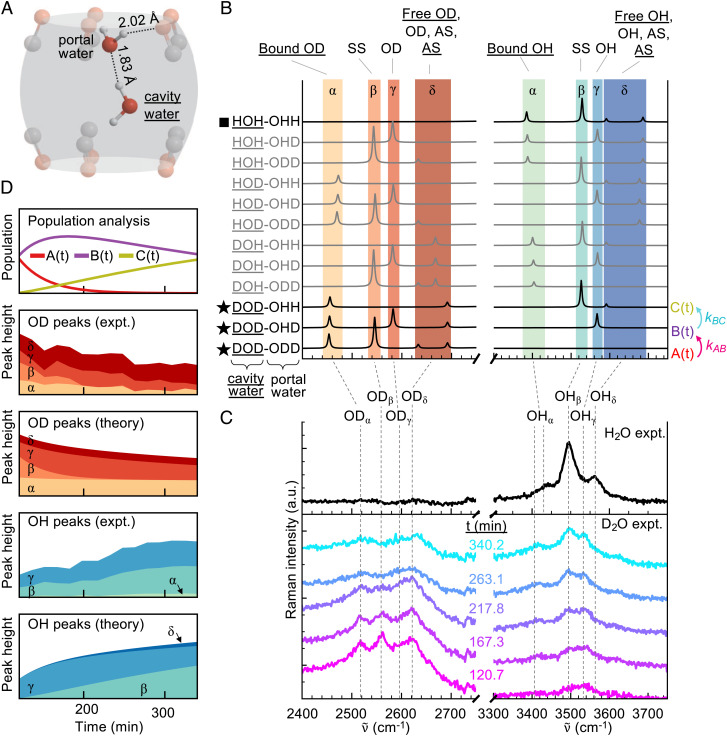
Confined water peak model from DFT and kinetic dynamics. (*A*) DFT-optimized geometry of water dimer inside CB[5]. (*B*) Normal mode frequencies for all 12 unique isotopologues of water dimer@CB[5]. Four OD bands ɑ–δ and four OH bands ɑ–δ are highlighted by colors that correspond with experimentally observed peaks. At *Top*, ɑ–δ bands are described by their vibrational origin (SS = ‘symmetric stretch’, AS = ‘asymmetric stretch’). Throughout the figure, underlined text relates to the cavity water. Isotopologues marked with a square are observed in the H_2_O experiment; those marked with a star are observed in the D_2_O experiment. (*C*) Experimental measurements of cavity-confined water from Fig. 2 *B* and *F* with Gaussian broadened peaks identified from (*B*). (*D*) Fitted experimental OD- and OH-band confined water dimer peaks compared to the same peaks from theory discussed in the text.

DFT is used extensively to model free water clusters, and inclusion of water clusters in CB has been shown to only slightly perturb their geometry (26). Therefore, a DFT-based model is used to verify that the observed narrow lines indeed come from a structured water dimer@CB[5] and to identify the individual modes. The optimized DFT of a linear water dimer@CB[5] Fig. 3*A*) produces a geometry in agreement with previous theoretical studies, giving a <0.03 Å difference in optimized hydrogen bond lengths compared to simulations of Biedermann et al. (22) (detailed comparison in *SI Appendix*, Table S8, coordinates reported in *SI Appendix*, Supplementary Note 2D). This geometry consists of a ‘portal water’ acting as a hydrogen-bond donor to the CB[5] electronegative carbonyls while also acting as a hydrogen-bond acceptor from a ‘cavity water’ sequestered inside the CB[5].

To explain the D→H isotopic exchange dynamics observed, the expected vibrational frequencies for all deuterium isotopologue variants of (H_2_O)_2_@CB[5] are calculated (Fig. 3*B*). Portal water hydrogens are interchangeable because their hydrogen bond lengths to respective carbonyls are essentially identical (*SI Appendix*, Table S8), yielding 12 unique isotopologues. This produces a wide fan of modes across the OD and OH bands. A labeling scheme is employed where underlining labels the cavity water, e.g., DOH-ODD represents a DOH cavity water hydrogen bound (through its H atom) to a D_2_O portal water. The relative peak heights and orderings from these frequency calculations can be simply explained as a perturbation of the carbonyl binding onto free water dimer modes (*SI Appendix*, Fig. S12) using a mass-spring model and DFT (*SI Appendix*, Fig. S13). Further supporting the validity of this model, the DFT calculations produce water-to-CB[*n*] peak ratios of the same order as observed in experiment (*SI Appendix*, Fig. S14).

To match with experimental data, the DFT modes are split into four frequency bands: ɑ–δ for both OD and OH (Fig. 3*B*, colored stripes) as noted in *SI Appendix*, Tables S2 and S3. In the H_2_O experiment (Fig. 3 *C*, *Top*), no deuterium exists in the system, so only HOH-OHH can be observed. The observed experimental spectrum of a strong central peak with two weak side peaks matches well with the DFT frequency calculation. Therefore, we assign the strong observed peak to the OH_β_ vibration and the low-intensity side peaks to the OH_ɑ_ and OH_δ_ vibrations. These bands are fit (details in *SI Appendix*, Supplementary Note 1 C.i) to OH spectra from five different locations on the sample (*SI Appendix*, Fig. S3), resulting in peak centers and FWHMs reported in *SI Appendix*, Table S2. The OH vibrations of (H_2_O)_2_@CB[5] observed are at lower frequencies (by 100–200 cm^−1^, *SI Appendix*, Tables S4 and S5) than in gas-phase molecular beam experiments (*SI Appendix*, Fig. S6), indicating that in general, hydrogen bonding is stronger for the CB-confined dimer than for the free dimer. This strong redshift is expected because the calculated O–O distances in the CB-confined dimer are much closer to those of ice than those of free water dimers (*SI Appendix*, Table S8 and Supplementary Note 2E). The extra hydrogen bonds between portal water and CB carbonyls mean that the vibrational frequencies of the CB-confined dimer overlap more closely with previously observed spectra for the water trimer (H_2_O)_3_ (*SI Appendix*, Fig. S9). The bands measured here are also broader, which could arise from inhomogeneities or the increased temperature relative to cryogenic experiments (*SI Appendix*, Supplementary Note 1D).

Three key observations help match the DFT bands to the D_2_O data: 1) Throughout the D_2_O experiment, 4 narrow peaks are seen in the OD region but only 3 narrow peaks in the OH region. This suggests that observed isotopologues have more deuterium than hydrogen. Hence, we assign peaks ɑ, β, ɣ, and δ inside the OD band, but only assign peaks ɑ, β, and ɣ inside the OH band. These peaks are fit (*SI Appendix*, Supplementary Note 1C.ii and Fig. S4) throughout the time of the experiment (*SI Appendix*, Fig. S5); fitting results are reported in *SI Appendix*, Table S3. 2) The OD_β_ peak disappears faster relative to the other OD peaks OD_ɑ_, OD_ɣ_, and OD_δ_. 3) Initially, the two OH peaks OH_β_ and OH_ɣ_ have roughly equal intensity, but after isotope exchange, the OH_β_ peak has higher intensity.

The simplest hypothesis explaining all three observations in the D_2_O experiment is that throughout the isotopic exchange, the states with DOD cavity water dominate the system and exchange occurs at the portal, i.e., the dominant states are DOD-ODD (initial state), DOD-OHD, and DOD-OHH (final state). This hypothesis explains 1) because these states have more deuterium than hydrogen. It also explains 2) because OD_β_ only appears for DOD-ODD, which is quickly removed from the system during the transition DOD-ODD → DOD-ODH. It explains 3) because OH_ɣ_ only appears for DOD-OHD, while OH_β_ only appears for DOD-OHH. Thus, under this model, the replacement of DOD-OHD by DOD-OHH will result in increasing OH_β_ peak intensity relative to that of OH_ɣ_.

The ability of this model to explain the D_2_O experiment reveals that the CB[5] cavity protects the cavity water from isotopic exchange, while the portal water is free to exchange isotopes with its surroundings. The CB[5] thus acts as a proton guard, gated by the portal water, and allows no access to the cavity water. This model both explains why a month of immersion in D_2_O is needed to deuterate the cavity with initial H_2_O and also explains experiments where D_2_O was incubated in CB[5] solution for shorter times. Incubating CB[5] in D_2_O for only 1 week or less yields spectra showing features of nonfully deuterated cavity water (*SI Appendix*, Fig. S2). The alignment of this model to experiments suggests that the dimer stays attached to one set of carbonyl portals without migrating to the other carbonyl portal because otherwise the portal water and cavity water would exchange roles. This is reasonable given the strong portal water-cavity water hydrogen bond (*SI Appendix*, Table S8) and the potential barrier required for the portal water to leave the electronegative environment near its carbonyl portals. The small accumulation of the OH_ɑ_ peak in experimental data arises from small fractions of cavity water becoming deuterated, representing a higher-order correction to our simple hypothesis. The modes from the isotopologue (D_2_O)_2_@CB[5] are also redshifted relative to previous spectra from free (D_2_O)_2_ (*SI Appendix*, Figs. S7 and S8 and Tables S6 and S7). For reference, the calculated vibrational frequencies for CB-confined dimers from DFT are compared directly with the experimental spectra in *SI Appendix*, Fig. S11.

To quantitatively verify the model for isotopic exchange in the D_2_O experiment, a simple rate equation model is used (Fig. 3*D*), which assumes that only the three isotopologues with the cavity water fully deuterated are observed experimentally, labeled as DOD-ODD = $A\left(t\right)$, DOD-OHD = B(t), and DOD-OHH = C(t). We assume that the system undergoes single D → H exchange according to rate constants $k_{\mathrm{AB}}$ and $k_{\mathrm{BC}}$. We use least-squares (*SI Appendix*, Supplementary Note 3A) to fit population dynamics to the experimental fitted peak heights of OD_ɑ, β, ɣ, δ_ and OH_ɑ, β, ɣ_ (Fig. 3*D*). The model uses Raman cross sections for each isotopologue from the DFT. The extracted dynamics show A(t) quickly decaying while B(t) slightly rises and then slowly decays. At the same time, C(t) grows slowly. The OD and OH peak heights generated from this kinetic model qualitatively match experiments well (Fig. 3*D*, theory vs. experiment) and thus support observations (1–3). The model curves predict a small OH_δ_ peak unresolved in the experiment, likely because it lies so close to the stronger OH_ɣ_ peak. No other combination of assumptions provides a suitable account of the data.

Fitted rate constants differ at different locations of the sample (which correspond to slightly different solid-state structures) but all display qualitatively similar behavior and support the same kinetic model for isotopic exchange (*SI Appendix*, Fig. S15*A*). For all locations, $k_{\mathrm{AB}}>k_{\mathrm{BC}}$ (*SI Appendix*, Fig. S15*B*). While $k_{\mathrm{BC}}$ is always approximately 10^−2^ min^−1^, $k_{\mathrm{AB}}$ has stronger variations and is an order of magnitude higher in locations iii and iv than locations i and ii. The concurrent shifts of the CB_450_ peak can also be fitted using the kinetic model in a consistent way across all four sample locations (*SI Appendix*, Fig. S16). This verifies that the isotopic water exchange and movement of the CB_450_ peak are correlated processes.

### Capabilities for Water Cluster Experiments.

**Fig. 4.**
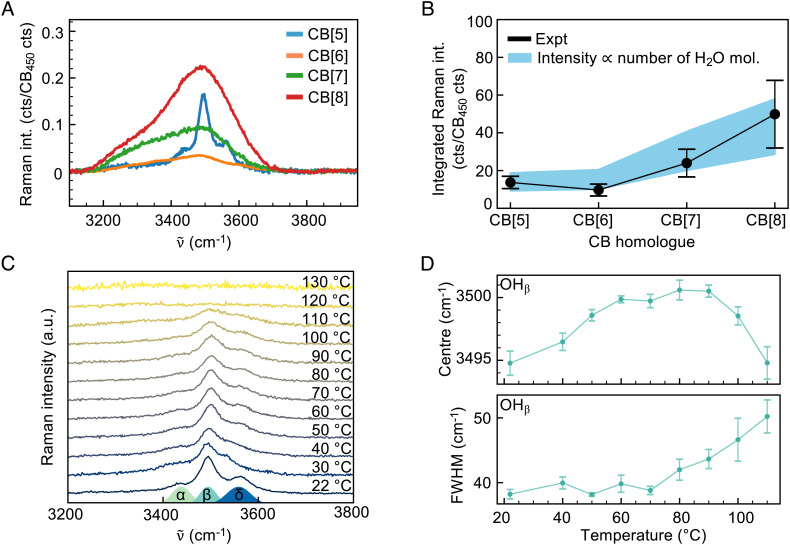
Capabilities enabled by a solid-state molecular capsule for water clusters. (*A*) Tuning CB[*n*] size changes degree of water confinement, resulting in differences in OH band broadening, with (*B*) more OH band signal for larger CB[*n*] homologues. Error bars are the SD from fitting normalized OH band areas over several trials, over different fitting parameters for the background subtraction. Model curve assumes that intensity is proportional to expected number of cavity-confined waters (22) divided by CB_450_ peak height found from DFT. Shading of model curve indicates uncertainty in the fit. (*C*) Solid-state system encapsulating water allows high-temperature studies of water clusters, with (*D*) OH_β_ peak features tracked as a function of temperature. Error bars are SD over four experimental trials.

A diverse range of fundamental studies on discrete water clusters, many of them difficult to access in existing systems, are enabled using cucurbituril nano-encapsulants. Three examples are provided here. First, the cluster size dependence: Various sizes of the CB[*n*] homologues can be used to reliably control the number of water molecules in the discrete clusters. This is shown in Fig. 4*A* where the effect of CB[*n*] cavity size is shown on the Raman spectra of the discrete water clusters, giving broader bands for the CB[6–8] homologues. The larger cavities provide more space for both varying water cluster shapes/orientations and increased fluctuations, leading to inhomogeneous broadening. Due to this broadening, the OH bands of CB[6–8] cannot be as easily assigned to specific vibrations as for the CB[5] but are still tentatively assigned to cavity-confined water clusters. The integrated OH band signal, normalized to CB_450_ peak height, increases as CB[*n*] size increases (Fig. 4*B*, solid line), which is expected as larger CBs hold more water. This behavior can be reproduced qualitatively using a simple model (Fig. 4*B*, blue shaded) which assumes that the overall OH band area, normalized to the calculated DFT intensity of the CB_450_ band, is proportional to the number of water molecules in the CB[*n*] cavity (taken from the prediction in ref. 22). The CB[6–8] broadened spectral distributions exhibit evidence of water clusters much less strongly networked than bulk H_2_O (*SI Appendix*, Fig. S10). Moreover, there are nonmonotonic changes in the spectral components representing networked vs. free water as *n* increases from 6 to 8 (*SI Appendix*, Supplementary Note 1F). Overall, the general agreement between experiment and prediction indicates that water clusters within the four major CB[*n*] homologues can be isolated and analyzed using Raman spectroscopy, enabling further studies on cluster dynamics for different numbers of water molecules.

Second, the temperature dependence: By incorporating a heating stage, temperature-dependent spectroscopy of this solid-state system is conducted from 20° C up to 130° C. Tracking the OH stretches of the (H_2_O)_2_@CB[5] (Fig. 4*C*) shows that the three main bands observed for the dimer at room temperature (OH_ɑ,β,δ_) shift and broaden with heating, ultimately disappearing completely at 120° C. This shows that superheated solid water dimers can be studied using our method, though further experiments and simulation will be required to fully elucidate the dynamics and energy barriers of these water dimers in the CB environment. The OH_ɑ_, OH_β_, and OH_δ_ peaks are fit over the temperature sweep. The strong central OH_β_ peak shows the most consistent trends across all measured sample locations, while OH_ɑ_ and OH_δ_ peak fits are noisy due to weaker signal (Fig. 4*D*).

As temperatures increase up to 80° C, the average hydrogen bonding strength experienced by the portal water from both the carbonyl portals and the cavity water decreases, causing its symmetric stretch peak OH_β_ to shift to higher frequencies. In this regime, the linewidth is unchanged. Hence, watching the temperature-dependent vibrational shift up to 80° C is a clear way to controllably decouple water molecules from their immediate environment and slowly weaken hydrogen bonds. It would clearly be interesting to tune the dimer temperature from 80° C to below room temperature to observe the sharp transition from weak hydrogen bonding to moderate/strong hydrogen bonding, where the potential becomes rapidly anharmonic and vibrational frequency rapidly decreases (39) and this is ongoing research. Above 80° C, the behavior is more complex and less clear. The frequency shift is reversed, now trending toward lower frequencies, and the mode starts to broaden with further temperature rise. Here, it appears that the portal water molecule gains enough energy to escape its confinement potential, enabling it to explore different configurations. This supercritically heated water dimer accesses more conformations and environments, broadening the OH line. The decrease in Raman shift requires further investigation to understand. The high frequency of these OH stretch vibrations would seem to preclude the usual decrease in Raman shift due to anharmonicity, though hydrogen-bonded stretches are much more anharmonic than typical stretches (39). The decrease in frequency could arise from some of the newly explored dimer conformations having relatively strong hydrogen bonding. These temperature-dependent observations suggest many further research directions, such as exploring hysteresis for different heating/cooling cycles at different rates, and the effects of surrounding solvents can now be explored.

Thirdly, chemical manipulation: CB[*n*]s enable diverse ways to actively manipulate the direct chemical environment of water clusters. To first demonstrate this, a mixture of CB[5]:Fe is used since Fe^3+^ ions are known to strongly bind to the CB portal carbonyls, acting as a capping agent (*SI Appendix*, Fig. S18*A*) (18). In this system, narrow bands from water clusters are still observed, but the bands are stronger and shifted to lower wavenumbers relative to those observed for uncapped CB[5] (*SI Appendix*, Fig. S18*C*). Moreover, the number of observed peaks decreases, suggesting that the original dimer becomes a monomer in the presence of Fe^3+^. While the D_2_O@CB[5] without Fe^3+^ shows D → H exchange on minute timescales (Fig. 3*C*), the D_2_O@CB[5] capped with Fe^3+^ exhibits narrower OD bands that persist over several hours in ambient atmospheres (*SI Appendix*, Fig. S18*D*). No isotope exchange was observed over >10 h showing that the confined dimer isotopic labeling can be protected even in hydrogen-rich environments. In addition, using larger CB[*n*] homologues also allows dynamics between host–guest chemistry and confined water clusters to be studied, as shown for the inclusion of ferrocene, which binds strongly into the CB[7] cavity interior and displaces cavity water (*SI Appendix*, Fig. S17). By engineering the CB[*n*] environment, it is thus possible to tune water cluster chemical interactions with their surroundings, which opens many possibilities and suggests that switchable systems can also be envisaged. Future work will explore the kinetics and thermodynamics of such capping with different sizes of CB[*n*] and with different additional guests in the cavity.

## Conclusions

In summary, drying CB[*n*] from aqueous solutions in a low-humidity environment allows water clusters to be studied using Raman spectroscopy, with narrow OD/OH bands observed for CB[5]. For these water@CB[5] clusters, analysis through DFT and kinetic modeling of isotopic exchange strongly suggests that the bands originate from a cavity-confined water dimer. This dimer consists of a ‘portal water’ which is hydrogen-bound to one CB[5] portal, while the ‘cavity water’ lies inside the CB[5] cavity. CB[5] cavity encapsulation slightly perturbs the free water dimer geometry, resulting in a compressed donor-acceptor hydrogen bond length in calculations and redshifted OH stretches in experiments. Our results suggest that the CB[5] cavity walls shield the inner cavity water, while the portal water readily interacts with its environment. The readily observed single-hydrogen bond connecting them is thus available for study. This system hence isolates a single hydrogen-bonded cavity water molecule protected by the CB[5] walls, while allowing for vibrational measurements at room temperature. Our observation of a CB[5]-confined water dimer in a ‘dry’ system provides an intriguing contrast to NMR results (40), which suggested that CB[5] cavity interiors are devoid of water whilst in solution. Because cavity-confined water can have such a drastic effect on cavity-binding energetics (40), it is important to further study how the presence of bulk water destabilizes CB[5]-confined dimers. While this first study lacks the resolution to discern fine features such as rotational-tunneling transitions (13, 41), it provides a valuable starting point for further measurement. Raman measurements from 200 to 700 cm^−1^ can access librational motions, key to hydrogen bond breaking/forming (42). Microwave measurements would be useful on CB-confined water to compare to the large body of previous work on high-resolution microwave spectra and fine structure of free water dimers (43). Although simple DFT calculations are sufficient here to identify water dimer vibrational bands and their physical origin, further work would incorporate more advanced theoretical methods on water clusters (44–47) using techniques such as flexible potentials (48, 49), anharmonic analysis (50, 51), and molecular dynamics.

This molecular capsule opens many accessible studies on an isolated water molecule and isolated hydrogen bond at ambient and higher temperatures. We show preliminary results verifying that the system gives versatile control over the water cluster environment, by modifying cavity confinement, temperature, and ion–water interactions. Ultrafast experiments on this system would also be interesting to probe the dynamics of intramolecular and intermolecular coupling within a single hydrogen-bonded system. Because the facile sample preparation only requires dissolving CB[*n*] in solution, developing complexity is straightforward, such as adding inclusion complexes or changing solution pH. This would enable spectroscopic studies of protonated water clusters, relevant to fundamental processes such as proton transfer. In larger CB[*n*] homologues with more degrees of freedom, we have initial results suggesting an interesting interplay between confined water clusters and confined gas molecules such as N_2_. Exploring such dynamics further is interesting for future studies of chemical reactions and gas capture. Moreover, an important step is to analyze these confined water clusters inside CB-assembled plasmonic nanocavities (52–54), relevant to plasmon hot carrier-induced water splitting and cavity-water strong coupling (55). Finally, comparing vibrational spectra of confined water in CB to those in other comparable supramolecular structures (56) would provide more information on how the water cluster geometry is perturbed by the cavitand.

## Materials and Methods

### Raman Measurements of CB[n] Solutions.

The molecules CB[5] and CB[7] are purchased from Sigma-Aldrich and used as received; CB[6] and CB[8] are synthesized in-house according to ref. 30. Raman spectra are collected with a Renishaw InVia Raman microscope with 1 mW of 633 nm excitation through a Nikon 50× objective (0.5 NA). For all experiments, multiple (3–4) sample locations are measured with representative spectra shown here (see SI for all scans). Spectra are collected from inside a home-built gas-flow cell through a glass coverslip unless noted otherwise. In a typical time-dependent Raman experiment, a concentrated (10 mM) solution of CB[5] (Sigma-Aldrich) in deionized water is drop-cast (100 µL) on a template-stripped gold substrate (as in ref. 53) and kept in the gas-flow cell under a gentle nitrogen flow, allowing excess water to evaporate (Fig. 1*A*). For high-temperature Raman measurements, the sample is placed in a Linkam HFS 91 temperature-controlled stage instead, and the temperature is gradually ramped at 10° C/h from room temperature to 130° C. Raman spectra are background corrected by subtracting a quadratic or cubic function fitting to the background data around the peaks of interest. Tracking multiple sample locations shows qualitatively similar behavior over time, so a single sample location is studied over time for CB[5] drying in H_2_O or D_2_O. The water cluster Raman shifts are independent of laser intensity and number of molecules, with different laser intensities and measuring different sample areas having no effect on Raman shifts. In principle, these measurements could also work in an infrared setup, which could provide complementary information to Raman spectroscopy. Raman spectroscopy is used here for practical benefits, namely easier sample preparation within a flow-cell setup, and potential of increasing signals in future studies using previously demonstrated surface-enhanced Raman spectroscopy in plasmonic nanocavities (52–54).

### Isotope Exchange and CB[n] Complexation.

For D_2_O experiments, 10 mM solutions of CB[5] in D_2_O (Sigma-Aldrich 99.9% D) are prepared and allowed to rest for 1 mo to ensure full isotope exchange of the cavity water in CB[5] (see *SI Appendix*, Supplementary Note 1B). For the CB[7]-ferrocene inclusion experiments, CB[7] solution is mixed with ferrocene (98%, Sigma-Aldrich) in a 1:1 molar ratio and sonicated for several hours. For the Fe^3+^ experiments, CB[5] is mixed with FeCl_3_ (Sigma-Aldrich, reagent grade, 97%) in a 1:2 CB:Fe molar ratio in deionized water and sonicated.

### DFT Calculations.

The Gaussian09 computational chemistry program was used to model water clusters through initial geometry optimization and then frequency calculations. Free water clusters were modeled with the B3LYP functional with 6-311++G(3dp, 3df) basis set, while water dimers inside CB[*n*] were modeled with the 6-31+G(d,p) basis set that can well-describe geometries and energies of simple water clusters with well-defined hydrogen bonds (57) (optimized geometries included in *SI Appendix*, Supplementary Note 2D).

## Supplementary Material

Appendix 01 (PDF)

## Data Availability

Data for all figures in main and supplemental texts are available online at: https://doi.org/10.17863/CAM.86770. Chemical coordinates of (H_2_O)_2_@CB[5] are reported in the *SI Appendix*.
