# Supplementary material for: Tracking water dimers in ambient nanocapsules by vibrational spectroscopy
Source: Proc Natl Acad Sci U S A. 2022 Dec 1;119(49):e2212497119. doi: 10.1073/pnas.2212497119 (PMC9894256; doi:10.1073/pnas.2212497119)
Supplement: Supplementary file 1 — Appendix 01 (PDF) [file pnas.2212497119.sapp.pdf]

PNAS

Supporting Information for
Tracking Water Dimers in Ambient Nanocapsules by Vibrational Spectroscopy

Alexander Y. Hwang[1,‡], Rohit Chikkaraddy[1], David-Benjamin Grys[1], Oren A. Scherman[*,2], Jeremy J. Baumberg[*,1], and Bart de Nijs[*,1]

[1] NanoPhotonics Centre, Cavendish Laboratory, Department of Physics, JJ Thompson Avenue, University of Cambridge, Cambridge, CB3 0HE, United Kingdom

[2] Melville Laboratory for Polymer Synthesis, Department of Chemistry, University of Cambridge, Lensfield Road, Cambridge, CB2 1EW, United Kingdom

‡Present address: Department of Applied Physics, Stanford University, Via Pueblo Mall, Stanford, CA, 94305, USA

*Corresponding Authors: Bart de Nijs, Oren A Scherman, Jeremy J. Baumberg

Email: BdN – bd355@cam.ac.uk, OS – oas23@cam.ac.uk, JB – jjb12@cam.ac.uk

This PDF file includes:

Supporting text (Supp. Note 1-6)
Figures S1 to S18
Tables S1 to S8
SI References

Supporting Information Text

Supplementary Note 1: Additional experimental details and analysis

1A. Influence of nitrogen atmosphere

An important element of our experiment is measuring Raman spectra in a nitrogen-rich atmosphere to isolate the signal contribution from cavity water. To show this, we conduct the "H_2O experiment" with CB[5] as described in the main text. After the sample has dried completely, the three main peaks (Fig. S1) attributed to OH_α, OH_β, and OH_δ as described in the main text are consistently observed while remaining in a nitrogen atmosphere. Because there is no deuterium in the system, no OD peaks are observed.

At a time point ~250 min, the nitrogen flow is switched off. On timescales of ~100 min the lineshapes do not change significantly, but after leaving the sample overnight we observe greatly broadened peaks where the original three-peak structure is not discernible. We attribute these broad peaks to interstitial water that has been filled by atmospheric water. We note that two previous works that report OH-band IR vibrational spectra of CB[6] (1), and CB[5], CB[8] (7) do not mention measuring the sample under nitrogen conditions, which may have made it difficult in those works to differentiate contributions from inner-cavity water and outer-cavity water.

At a time of ~4500 minutes, we turn the nitrogen flow back on and find that the structured water dimer peaks are immediately recovered, indicating that the broad peaks come from weakly bound water in the crystal that can immediately be swept away by nitrogen flow. The broad peaks come back again eventually if the nitrogen flow is turned off.

1B. Slow incorporation of deuterium into CB[5]

Initial experiments show an interesting relationship in the CB[5] "D_2O experiment" between the structured water peaks seen in the OD/OH bands and the incubation time of CB[5] in D_2O (in ambient conditions).

If CB[5] is dissolved with D_2O, and the solution immediately dried for measurement on the same day, no OD peaks are observed, and three clear OH peaks are observed (Fig. S2b). These three clear OH peaks have the same lineshape and Raman shift as those (*i.e.* OH_α, OH_β, and OH_δ) measured in the "H_2O experiment" without any deuterium in the system. This suggests that timescales less than one day are not sufficient to exchange a significant number of cavity hydrogens with deuteriums.

If CB[5] is incubated for 1 week in D_2O, structured peaks in the OD band (Fig. S2c) start to emerge. However, these peaks are different than those observed for the sample analysed in the main text (~1 month incubation time, Fig. 3c). After 1 month incubation, four OD bands OD_α (~2515 cm^{-1}), OD_β (~2560 cm^{-1}), OD_γ (~2600 cm^{-1}), and OD_δ (2620 cm^{-1}) are visible (Fig. 3c). However in the 1 week incubation sample no OD_α peak near 2515 cm^{-1} was present. The furthest downshifted peak was the OD_β peak near 2560 cm^{-1}. The absence of the OD_α peak implies that at early times in the experiment, the sample could either have cavity <u>DOH</u>- or <u>HOH-</u> (Fig. 3b).

Another major difference is that in the 1 month incubated sample, at long experimental times after the majority of isotopic exchange has been completed, the OH peaks still do not completely resemble those from the H_2O experiment. We use this information to conclude that the cavity <u>DOD</u>- in the 1 month incubated sample is relatively robust to exposure to hydrogens in its environment and resists isotopic exchange. Contrastingly, in the 1 week incubated sample, after hour timescales, the system reproduces the OH band triplet of the H_2O experiment (OH_α, OH_β, and OH_δ). This suggests that in the 1 week incubated sample, isotopic exchange can quickly drive the cavity waters to <u>HOH</u>-OHH. A consistent explanation for this hypothesis would be that in the 1 week incubated sample, we have a transition from initial states with majority <u>HOH</u>-ODD and <u>HOH</u>-OHD to final states of majority <u>HOH</u>-OHH. Timescales of 1 week seem to be insufficient to replace <u>HOH</u> cavity waters with deuterium.

1C. Fitting procedure and detailed results
i. H_2O experiment

For the five different sample locations tracked over time in the CB[5] H_2O experiment, OH spectra are averaged over the 'cavity-confined' region and shown in Fig. S3. Each shown spectrum has been background-subtracted using a cubic polynomial. The resulting curves are then fit to:

- 4 broad Gaussians with fixed (peak centre [cm^{-1}], FWHM [cm^{-1}]) = (3280, 141.3), (3370, 117.75), (3466, 164.85), (3605, 101.265). Using 3-4 broad Gaussians is common practice to fit bulk-like confined water OH spectra (8, 9); these peaks represent a background of residual bulk water in the system.
- 3 narrow Gaussians OH_α, OH_β, OH_δ with peak centre, FWHM, and height as fitting parameters.

The mean and standard deviations of fitted peak centres and FWHMs over these five different sample locations are reported in Table S2. Overall the spectra and fitted parameters are consistent over the five different sample locations.

ii. D_2O experiment
For each sample location tracked over time in the CB[5] D_2O experiment, OD and OH spectra are background-subtracted by a cubic polynomial. For the curve fits extracted from the data in Fig. 2,3 of the main text, see Fig. S4. Then the curves are fit to:

- 8 broad Gaussians with fixed (peak centre [cm^{-1}], FWHM [cm^{-1}]) = (2280, 131.8), (50, 117.8), (54, 127.2), (2595, 170), (3280, 141.3), (3370, 117.75), (3466, 164.85), (3605, 101.265). Akin to the H_2O experiment, these broad Gaussians represent residual background bulk-like D_2O, H_2O, and HOD in the system.
- 7 narrow Gaussians OD_α, OD_β, OD_γ, OD_δ, OH_α, OH_β, and OH_γ. OH_δ is not fitted because it cannot be observed in the experimental data, and attempting to fit it results in erratic results. There is a broad shoulder visible near 3600 cm^{-1}, but we do not fit it as it is weak, located at the same position as a broad peak in bulk water, and cannot be explained by our current theory as it is more blueshifted than the OH_δ peak in the H_2O experiment. Each narrow Gaussian has fixed FWHM (specified in Table S3) in the fitting routine to reduce degrees of freedom in the fit. Peak heights are fitted, which are reported in Fig. 3d of the main text. Peak centres are allowed to vary within the fit routine (±15 cm^{-1} for $OD_{\alpha-\delta}$, ±20 cm^{-1} for OH_α, ±10 cm^{-1} for OH_γ and OH_δ), but actual fitted peak centres vary on the order of a few cm^{-1} (Fig. S5). The mean and standard deviations of these fitted peak centres over time are reported in Table S3.
- The frequency range near the small peaks at 2750 cm^{-1} is excluded from the fit. The peaks in this range do not evolve over the course of the experiment and are present in non-deuterated samples, meaning they do not represent any OD stretches.

1D. Comparison of CB[5]-confined water dimers to free water dimers in literature

i. $(H_2O)_2$

The OH spectra for the confined water dimer $(H_2O)_2$@CB[5] from this work are compared against OH spectra for benzene-bonded and free gas-phase water dimers from the molecular beam literature in Fig. S6. The D-B, A-S, and A-AS/D-F stretches correspond to the peak labels from this work OH_α, OH_β, and OH_δ, respectively. Notably, the free water dimer band assignments are still under debate—though most papers from Huisken et al. (11) onwards agree that the donor bonded stretch (D-B) is located near 3600 cm^{-1}, the recent study by Zhang et al. (15) found D-B around 3533 cm^{-1}. The frequencies reported by the past experiments are compared to the CB[5]-confined dimer results in Table S4. Moreover, the shift reported here for $(H_2O)_2$@CB[5] mode frequencies relative to past observed free dimer modes are shown in Table S5 (because no distinction could be made between D-F and A-AS in these experiments, a range of potential shifts are listed).

The CB[5]-confined water dimer bands are all found to be shifted to lower frequencies than the free water dimer bands, meaning that in general, hydrogen bonding is stronger for the CB-confined dimer than for a free dimer. This is consistent with the shorter donor-acceptor hydrogen bonding length for CB-confined dimers than for free dimers computed in this work and by others (Supp. Note 2.E). The D-B and A-S modes are still both visible in the $(H_2O)_2$@CB[5] system and appear to redshift approximately the same amount relative to experiment (Table S5). Meanwhile, the D-F and A-AS modes cannot be distinguished from each other in our experiment, suggesting they shift by differing amounts when going from free water dimer to CB-confined dimer. This can be explained by a mass-spring model (Fig. S13). In the mass-spring model, starting with a free water dimer eigenfrequencies, then increasing the acceptor-carbonyl and acceptor-donor H-bond strengths together (Fig. S13d) results in both a quick merging of the D-F and A-AS modes and a uniform redshift of all the modes, as seems evident in the experiment. While past experimental OH spectra for the isolated reference of a water molecule bound to two carbonyls could not be found, past theoretical calculations verify that a linear water dimer bound to two carbonyls may have significant redshifts >100 cm^{-1} (16).

In addition, observed bands in the CB[5]-confined dimer are broader than in previous studies with molecular beams. This could come from inhomogeneous variations of CB cavity orientation/external strain within the solid-state structure probed by the focal volume of the incident laser beam, as well as a homogeneous contribution from water dimers being more free to thermally diffuse at room temperature compared to the cryogenic temperatures of molecular beam experiments. OH stretch vibrations coupling to CB cavity vibrational modes would also lead to homogenous broadening.

ii. $(D_2O)_2$

Unlike in the H_2O experiment, observed OD bands in the CB[5] D_2O experiment are the result of several different deuterated dimer isotopologues (Fig. 3, Fig. S4, Fig. S11). However, based on our modelling (Fig. 3b,c) we can still identify the locations of the fundamental $(D_2O)_2$@CB[5] dimer vibrations within our measurement. The fundamental vibrations D-B, A-S, and A-AS/D-F correspond to the peak labels from this work OD_α, OD_β, and OD_δ, respectively. In Fig. S8, we compare these fundamental vibrations to those from molecular beam $(D_2O)_2$ experiments in literature; absolute frequency values from literature are collected in Table S6.

Qualitatively, the results are very similar to those in the case of the $(H_2O)_2$ dimer when compared to literature (Fig. S6). The encapsulation of the deuterated dimer shifts vibrational modes to lower frequencies compared to the molecular beam water dimer experiments. However, a subtle difference in these encapsulation-induced redshifts is observed between $(H_2O)_2$ and $(D_2O)_2$. To illustrate, the mode frequencies for D-B, A-S and Free OH stretches as reported for both $(H_2O)_2$ and $(D_2O)_2$ by Otto et al (14) (tabulated in Table S4 and S6) are plotted Fig. S8 (bottom row). Here, the OD stretch frequencies are scaled by 1.369, the ratio of H_2O:D_2O monomer stretch frequencies (3657:2672 cm^{-1}) from Otto et al (14). The $(H_2O)_2$ and $(D_2O)_2$ frequencies are

compared to same modes at lower frequencies of $(H_2O)_2$@CB[5] and $(D_2O)_2$@CB[5] (Fig. S8, top row). After the frequency rescaling, the D-B and Free OH modes of both $(H_2O)_2$ and $(D_2O)_2$ shift nearly identically upon CB[5] encapsulation (graphically, the slopes of the arrows in Fig. S8 are the same between $(H_2O)_2$ and $(D_2O)_2$). However, the A-S mode is observed to shift differently. The differing slopes of the arrows in Fig. S8 indicate that frequency rescaling alone cannot explain the A-S encapsulation redshift difference between $(H_2O)_2$ and $(D_2O)_2$. This difference in A-S mode frequency shifts may point to slightly different water-carbonyl interactions between $(H_2O)_2$ and $(D_2O)_2$, though it is hard to draw conclusions from the limited data available in the present study.

1E. Comparison of $(H_2O)_2$@CB[5] to free water trimers in literature

In the previous section we showed that $(H_2O)_2$@CB[5] OH stretches were redshifted relative to those of free water dimers. While the free $(H_2O)_2$ dimer only has a single 'donor' water molecule, $(H_2O)_2$@CB[5] has two 'donors' because the portal water hydrogen bonds to the CB's carbonyls . Therefore, we also include a qualitative comparison of observed $(H_2O)_2$@CB[5] vibrations to previous water trimer–$(H_2O)_3$ –spectra from literature (Fig. S9). Because the water trimer contains an additional H-bond acceptor, observed bonded OH bands shift from ~3530-3600 cm^{-1} in the dimer ~3400-3550 cm^{-1} in the trimer, which is also where $(H_2O)_2$@CB[5]'s vibrations are found. This shows qualitatively how adding the H-bond acceptor to the dimer can shift substantially shift modes to lower frequency. However, detailed comparisons between $(H_2O)_2$@CB[5] and the free water trimer are difficult because the water trimer's ground state is cyclic (20) as compared to the linear structure of the water dimer.

1F. Water cluster vibrations as a function of CB size

To explore in-depth the effect of changing CB[*n*] size on their confined water clusters, we plot water cluster@CB[5-8] OH spectra compared to a Raman spectrum taken on the same system of liquid H_2O in Fig. S10. All the CB[*n*] OH spectra are peaked at nearly the same value, near the OH_β peak (portal water symmetric stretch) of $(H_2O)_2$@CB[5]. This indicates for water clusters in all the CB[*n*] cavities, the geometry is anchored through a hydrogen bond from the portal water to the CB carbonyl portal. This finding is consistent with optimized geometries for CB[5-8] from literature(1).

CB[6-8] have much broader spectra than CB[5], because the confined water clusters are more free to scramble their orientation and behave like bulk liquid water, which is also consistent with literature(4). At the same time, the broad CB[6-8] spectra all appear markedly different from the Raman spectrum of bulk liquid water. The CB[6-8] spectra are shifted to higher frequencies compared to bulk water, and their frequency distributions are more strongly weighted towards the higher frequency portion. Both observations indicate that the CB[6-8]-confined water clusters have overall weaker hydrogen bonding (*i.e.* fewer H-bonds per water molecule) than bulk water, as their OH stretches are not as downshifted relative to the free OH vibrational frequency. These results are similar to those obtained in previous studies on confined water inside mesoporous silica(8, 9), where adding confinement shifted OH spectra to higher frequencies.

The CB[6-8] spectra show an interesting non-uniform dependence of spectral distribution as a function of *n*. The low-frequency spectral shoulder from 3200-3400 cm^{-1} signal (representing relatively networked water with more average H-bonds) is larger in CB[7] compared to CB[6], but smaller in CB[8] compared to both CB[6] and CB[7]. As shown in Fig. S10, this area corresponds to the free $(H_2O)_{4-5}$ bonded Raman vibrations measured by Otto *et al*.(14) Meanwhile, the high-frequency spectral shoulder from 3500-3600 cm^{-1} (representing relatively free water with fewer average H-bonds) increases monotonically as CB size is increased from CB[6] to CB[7] to CB[8]. These two observations suggest that water clusters inside the CB[6-8] cavities consist of a more strongly networked-component that is most prominent in CB[7] and a weakly-networked component that becomes more prominent as cavity size increases. More detailed experiments on the water clusters in these larger cavities are clearly interesting for further study while more sophisticated theory than the simple DFT used here to study the dimer would be desirable to account for molecular dynamics.

1G. Experimental dimer spectra compared to DFT lines

In Fig. S11 we make a direct comparison between experimental spectra in the CB[5] H_2O and D_2O experiments and vibrational lines calculated with DFT methods outlined in methods section of the main text and Supp. Note 2. Calculated DFT peak positions vary from experimentally measured peaks by ~50 cm^{-1} or less. This order of discrepancy between theory and experiment is also reflected in recent water dimer vibrational frequency calculations (13, 15) that employ more sophisticated theory/basis sets (HF, MP2, M06-2X, and CCSD(T)) than our study. Our study's simple DFT has the additional complication of having the water dimer trapped within a complex molecule, which also makes aligning theoretical lines with experimental measurements more difficult.

Supplementary Note 2: DFT simulations of free and confined water monomers and dimers
2A. OH stretches of free water dimers

Water monomers support two normal stretching modes (Fig. S12a,b): the symmetric stretch (SS, lower frequency) and anti-symmetric (AS, higher-frequency) stretch.

DFT shows that the modes of a free water dimer consist of two weakly coupled sets of these monomer normal modes. We first optimise a free water dimer geometry (Fig. S12c), resulting in a linear hydrogen bond between donor (D) and acceptor (A), as expected from literature (20, 22). The DFT-calculated frequency spectrum has four normal stretching modes as expected (Fig. S12d, orange). Importantly, the eigenmodes corresponding to the four calculated eigenfrequencies reveal two weakly coupled sets of AS and SS stretches, one set based on the donor molecule (D-SS, D-AS) and one based on the acceptor molecule (A-SS, A-AS). All four vibrations exhibit a shift to lower frequency from the monomer vibrations because the hydrogen bond delocalizes electron density from the electronegative oxygen. The donor vibrations shift more strongly (25-80 cm^{-1}) than the acceptor vibrations shift (7-10 cm^{-1}) because the donor vibrations have hydrogen, a light atom, participating in the hydrogen bond. The small vibrational shift of the acceptor molecules is notable in our study because our observed linewidths range from 20-60 cm^{-1}, meaning that it would be difficult to distinguish how many cavity water dimers are present *vs* singly-occupied cavities with a just a portal (acceptor) water. Finally, because the hydrogen bond affects polarizability of donor stretches more than of acceptor stretches, the donor stretches have higher Raman intensity than the acceptor stretches.

2B. Comparing DFT of CB-confined water dimer with mass spring model

As stated in the main text, to expand upon the DFT for a free water dimer, we add the linear water dimer to the inner cavity of CB[5] and optimize the geometry to the B3LYP/6-31+G(d,p) level of theory. The water cluster geometry (Fig. 3a, main text) strongly resembles the free water dimer and qualitatively matches those in references [(2, 4)].

However, the $(H_2O)_2$@CB[5] geometry has some important differences from that in free $(H_2O)_2$. First, the acceptor water molecule now weakly hydrogen bonds to the electronegative carbonyl portals on one side of the CB. Hence this molecule is labelled the 'portal water'. The donor water molecule lies inside the CB[*n*] cavity, so we label this molecule the 'cavity water'. In addition, the hydrogen bond length between donor and acceptor decreases relative to that of free $(H_2O)_2$, which is consistent with literature[1,6].

The extra bonds on the acceptor water and shorter hydrogen bond between donor and acceptor redshift the OH stretch modes, as seen in the DFT eigenfrequency calculation (Fig. S13a). In addition, the A-SS mode now has the highest Raman intensity because it has two hydrogens involved in hydrogen bonds instead of just one. Finally, the mode ordering between free $(H_2O)_2$ and $(H_2O)_2$@CB[5] slightly changes, *i.e.* the modes in order of increasing frequency are [D-SS, D-AS, D-AS, A-AS] for free $(H_2O)_2$ and [D-SS, D-AS, A-AS, D-AS] for $(H_2O)_2$@CB[5].

The above qualitative differences in the DFT frequency calculation for free dimer vs. CB[5]-confined dimer can be explained by considering a simple mass-spring model for the water dimer system (Fig. S13b). In the mass-spring model, consider the unperturbed covalent OH bond to have spring constant k, but the H-bond within the dimer adds a perturbation of p and ℓp to this spring constant for donor and acceptor, respectively. To illustrate the point, we let $p = 0.1k$ and $\ell = 0.1$, because the acceptor spring constants should be more weakly affected by the perturbation than the donor springs. Moreover, we introduce the spring constant perturbation q that comes from the acceptor-carbonyl H-bond. Finally, the molecules are coupled through a weak spring with spring constant $r = 0.2p$.

First, we isolate the effect of the acceptor-carbonyl H-bond (Fig. S13c), slowly increasing its bond strength by increasing the factor q/p. Increasing the acceptor-carbonyl H-bond strengths makes the anti-symmetric modes merge and cross before the symmetric modes (Fig. S13c), which explains the change in mode ordering we observe in DFT. Then, we not only increase the acceptor-carbonyl H-bond strength but also simultaneously increase the dimer intermolecular H-bond strength (Fig. S13d). This is done by introducing the parameter $t = [0,1]$ and parametrizing the acceptor carbonyl H-bond strength $q(t) = pt$ and the dimer H-bond strength $p(t) = (0.1 + 0.1t)k$. Adding in this effect retains the mode crossing/re-ordering of the anti-symmetric modes obtained previously and also causes all the modes to shift to lower frequency, which was observed in the DFT eigenfrequency calculation and in the experiment.

2C. Intensity ratios between water and CB[n] vibrations in CB-confined water dimer

As one check on the validity of our DFT-calculated results for the confined water dimer in CB[5], we consider the experimentally-observed water cluster Raman intensities divided by the Raman intensity of the CB_{450} and CB_{830} vibrations (Fig. S14). We compare these intensity ratios to intensity ratios of DFT-calculated water dimer vibrations to DFT-calculated CB_{450} and CB_{830} vibrations. We find that the intensity ratios for experiment and theory lie in the same order of magnitude (~0.01), which provides support that our DFT calculations are reasonable to support our experimental results.

2D. DFT-optimized geometry of $(H_2O)_2$@CB[5]

We report the DFT-optimized geometry of $(H_2O)_2$@CB[5] using B3LYP/6-31+G(d,p)

96

C30N20H34O12

C	-2.330049000	-2.412488000	2.034326000
N	-1.706075000	-3.360036000	1.231802000
C	-2.440266000	-3.627624000	0.011823000
C	-3.586301000	-2.547880000	0.033953000
N	-3.425633000	-1.917871000	1.323985000
N	-1.764232000	-3.368449000	-1.245147000
C	-2.295827000	-2.284978000	-1.921910000
N	-3.307011000	-1.740884000	-1.143944000
O	-2.019918000	-2.107343000	3.166990000
O	-1.961005000	-1.893115000	-3.029424000
C	-0.568119000	-4.120909000	1.720702000
C	-0.619225000	-4.102542000	-1.761783000
C	1.511868000	-2.878024000	-2.036098000
N	0.668744000	-3.673986000	-1.266456000
C	1.296437000	-4.130517000	-0.040116000
C	2.694833000	-3.409699000	-0.055356000
N	2.657914000	-2.644026000	-1.287362000
N	0.715098000	-3.702336000	1.209257000
C	1.573023000	-2.896952000	1.958821000
N	2.716479000	-2.686633000	1.195119000
O	1.307116000	-2.509437000	-3.176841000
O	1.387523000	-2.526647000	3.098938000
C	3.721600000	-1.799080000	-1.780013000
C	-2.950679000	1.388340000	2.070528000
N	-2.807957000	2.582020000	1.369072000
C	-3.520399000	2.585738000	0.110690000
C	-4.221969000	1.178404000	0.094527000
N	-3.677462000	0.513639000	1.264458000
N	-2.739266000	2.586483000	-1.114910000
C	-3.055333000	1.509614000	-1.937815000
N	-3.865839000	0.644556000	-1.200247000
O	-2.552388000	1.166149000	3.195296000
O	-2.740030000	1.368298000	-3.102060000
C	3.154013000	0.601415000	-1.924749000
N	3.739369000	-0.449999000	-1.245806000
C	4.395632000	-0.030612000	-0.018819000
C	4.120170000	1.520902000	0.022059000
N	3.355830000	1.756327000	-1.190219000
N	3.850276000	-0.502484000	1.233847000
C	3.319972000	0.521907000	2.011644000
N	3.441070000	1.704920000	1.283360000
O	2.595589000	0.536423000	-3.010839000
O	2.908609000	0.423703000	3.148505000
C	2.895628000	3.045379000	-1.672514000
C	0.450458000	3.369586000	-1.947425000
N	1.604106000	3.464366000	-1.172697000
C	1.375006000	4.117787000	0.099398000
C	-0.183652000	4.329699000	0.118861000
N	-0.613247000	3.798900000	-1.159258000
N	1.627422000	3.353443000	1.302253000
C	0.477346000	3.139763000	2.052910000
N	-0.600935000	3.622158000	1.310756000

O	0.394126000	3.031787000	-3.113190000
O	0.421239000	2.665871000	3.169188000
C	3.816140000	-1.880042000	1.685349000
C	2.934562000	2.957718000	1.796945000
C	-1.941804000	3.635332000	1.858112000
C	-1.983337000	3.720642000	-1.621153000
C	-4.169959000	-0.689785000	-1.666591000
C	-4.150407000	-0.755837000	1.791426000
H	-2.821588000	-4.659257000	0.019259000
H	-4.591138000	-2.987027000	-0.049171000
H	-0.540897000	-3.976614000	2.803950000
H	-0.722265000	-5.183757000	1.488784000
H	-0.608494000	-3.948241000	-2.843417000
H	-0.762316000	-5.167364000	-1.538378000
H	1.380543000	-5.228231000	-0.058607000
H	3.538612000	-4.116018000	-0.091016000
H	3.579594000	-1.713906000	-2.860043000
H	4.689296000	-2.271422000	-1.567567000
H	-4.237653000	3.420151000	0.092634000
H	-5.316942000	1.247336000	0.182305000
H	5.466771000	-0.273822000	-0.077468000
H	5.042017000	2.120696000	-0.002959000
H	2.799339000	2.967435000	-2.758179000
H	3.649073000	3.802253000	-1.419019000
H	1.936018000	5.064237000	0.135740000
H	-0.470107000	5.389784000	0.195272000
H	3.718643000	-1.846293000	2.773331000
H	4.761566000	-2.366394000	1.411232000
H	3.652912000	3.758130000	1.576061000
H	2.835887000	2.834270000	2.878449000
H	-1.850996000	3.488376000	2.937368000
H	-2.408113000	4.607046000	1.650894000
H	-1.942684000	3.615043000	-2.708319000
H	-2.506688000	4.649214000	-1.355644000
H	-4.042910000	-0.676733000	-2.751856000
H	-5.210428000	-0.933228000	-1.417065000
H	-4.016894000	-0.721968000	2.875727000
H	-5.214366000	-0.874183000	1.549399000
O	-0.124265000	0.134709000	-2.008622000
H	-0.716215000	-0.364644000	-2.595248000
H	0.635474000	0.368688000	-2.568838000
O	0.699300000	-0.300225000	0.612383000
H	0.113632000	-0.010595000	1.322924000
H	0.257423000	-0.126636000	-0.244554000

2E. Comparison of geometry to literature

The DFT-optimized geometry of $(H_2O)_2$@CB[5] computed in this study is compared to those obtained by other studies in literature in Table S8. Covalent OH bond lengths match within .003 Å to Biedermann et al. (4) but hydrogen bond lengths vary slightly more: within .03 Å for intra-dimer hydrogen bonds and within .1 Å for dimer-carbonyl hydrogen bonds.

O-O distances between water molecules are a commonly used metric to compare strengths of hydrogen bonding. The computed O-O distances (2.782-2.789 Å) for the CB-confined water dimer are shorter than the 2.912 Å O-O distance(23, 24) predicted for free water dimers that many advanced CCSD(T) theoretical methods have converged upon. This means that CB-confined dimers are more tightly hydrogen-bound than in the free gas phase, which verifies why their OH spectra are shifted to lower frequencies compared to gas phase dimers (Fig. S9). In fact, the O-O distances in the CB-confined dimer are comparable to those observed in ice (2.75 Å(25)) and are in the range of O-O distances reported for liquid water (2.73-2.89 Å(26)), again justifying the large spectral shifts we observe in CB-confined dimers relative to gas-phase dimers.

Supplementary Note 3: Kinetic model for isotopic exchange

3A. Model

We develop a simple rate equation model to explain the isotopic exchange of the D_2O experiment presented in the main text. We assume three possible states in the system, $\{A(t), B(t), C(t)\}$ corresponding to the populations of {DOD-ODD, DOD-OHD, DOD-OHH}, respectively (Fig. 3b). We assume that the system undergoes exchanges of single hydrogen atoms to deuterium atoms according to rate constants k_{AB} and k_{BC}, which represent the exchange from $A \rightarrow B$ and $B \rightarrow C$, respectively.

The rate equations are then:

$$\begin{cases} \dfrac{dA(t)}{dt} = -k_{AB}A(t) \\ \dfrac{dB(t)}{dt} = k_{AB}A(t) - k_{BC}B(t) \\ \dfrac{dC(t)}{dt} = k_{BC}B(t) \end{cases}$$

These are equivalent to:

$$\frac{d\boldsymbol{y}}{dt} = \boldsymbol{K}\boldsymbol{y}$$

$$\boldsymbol{K} = \begin{pmatrix} -k_{AB} & 0 & 0 \\ k_{AB} & -k_{BC} & 0 \\ 0 & k_{BC} & 0 \end{pmatrix}, \boldsymbol{y} = \begin{pmatrix} A(t) \\ B(t) \\ C(t) \end{pmatrix}$$

The solution to the initial value problem is then:

$$\boldsymbol{y}(t) = \exp(\boldsymbol{K}t)\,\boldsymbol{y_0}$$

We then use least-squares to minimize the quantity:

$$|\boldsymbol{ExptData} - \boldsymbol{IsotopeTransform} \cdot \boldsymbol{y}^{*}(t)|$$

where $\boldsymbol{ExptData}$ contains the fitted OD and OH peak heights for each time (Fig. 3d ‘expt’), $\boldsymbol{IsotopeTransform}$ contains the OD and OH peak heights for each state from DFT (Fig. 3b), and $\boldsymbol{y}^{*}(t)$ is the solution to the initial value problem for fitted values of $\boldsymbol{y_0}$, k_{AB}, and k_{BC}.

3B. Kinetic model results for different sample locations

In the CB[5] D_2O isotopic exchange experiment, four sample locations were tracked over time and all exhibited qualitatively similar behavior (Fig. S15). In general, the features observed over all locations support the kinetic model for D→H exchange developed in the main text. All four locations' population dynamics of A (DOD-ODD), B (DOD-OHD), and C (DOD-OHH) show a fast exchange from $A \rightarrow B$ followed by a slower exchange from $B \rightarrow C$. The initial fast exchange from $A \rightarrow B$ manifests in the fast disappearance of the OD_β mode in each kinetic model result. Moreover, all samples show the slow replacement of OH_β peaks with OH_α peaks over time, consistent with the full replacement of deuterium with hydrogen in the portal waters. Samples iii and iv show much faster dynamics than those of samples i and ii, likely owing to a different solid-state structure in these locations that leaves water@CB[*n*] more exposed. These faster dynamics are not completely resolved in our experiment, likely leading to the slightly larger discrepancy between experiment and theory.

Supplementary Note 4: Fitting CB[5] peaks to the kinetic model

The CB_{450} (Fig. S16a) and CB_{830} (Fig. S16c) peaks shift consistently across four sample locations during the isotopic exchange. CB_{450} shifts to lower frequencies while CB_{830} shifts to higher frequencies. Here we use results from the kinetic model to explain the shift of these CB[5] vibrations.

We assume the population dynamics of the kinetic model from the previous section and assume each state $\{A(t), B(t), C(t)\}$ exhibits CB[5] frequency shift of $\tilde{\nu}_i^{450}$ and $\tilde{\nu}_i^{830}$, $i \in \{A, B, C\}$. Given these assumptions, we fit the overall CB[5] frequencies of the system to experimental curves (Fig. S16a,c, dashed lines). The CB_{450} peak produces the same fitted frequency ordering across all four locations (Fig. S16b): $\tilde{\nu}_{\underline{DOD}-OHH}^{450} < \tilde{\nu}_{\underline{DOD}-ODD}^{450} < \tilde{\nu}_{\underline{DOD}-ODH}^{450}$. This suggests that isotopic exchange of water and the movement of the CB_{450} peak are correlated. The CB_{830} peak produces less consistent results (Fig. S16d).

Supplementary Note 5: Cavity water removal by ferrocene in CB[7]

If the OH band peaks we report in CB[7] come from cavity-bound water, then the peaks should decrease in amplitude when a guest molecule binds inside CB[7] and expels cavity water. We use ferrocene (Fc), which binds strongly inside the CB[7] cavity to verify this behaviour.

Two of the distinctive powder Raman peaks of ferrocene lie at ~310 cm^{-1} (Fc_{310}, symmetric Fe-Cp ring stretch) and ~1100 cm^{-1} (Fc_{1100}, Cp ring breathing) (Fig. S17a, black curve), which agrees with literature (27, 28). On the other hand, solidified CB[7] in a nitrogen environment has no major peaks near 310 cm^{-1} and 1100 cm^{-1} (Fig. S17a, yellow-orange curves). As expected, the CB[7] does show strong CB_{450} and CB_{830} peaks.

A Fc@CB[7] inclusion complex is formed by mixing Fc and CB[7] solutions in a 1:1 molar ratio, then the solution is dried in a nitrogen environment (same procedure as for CB[7] solution alone) (Fig. S17a, blue-green curves). In this case, a slight shift of the Fc_{310} peak distributions to higher frequencies is observed, which we attribute to higher force constants as a result of CB-Fc complexation(28). In addition, the CB_{450} and CB_{830} peaks shift to lower frequencies by ~2-3 cm^{-1}, which also strongly agrees with literature (28). Finally, the Fc_{1100} mode sees virtually no shift upon CB[7] inclusion, which is again reflected in literature (28). Thus the Raman spectrum of our Fc@CB[7] inclusion solid strongly suggests Fc resides inside the CB[7] cavity.

To analyse the effect of the Fc inclusion on the OH band, the background of the OH-band is fitted using a cubic polynomial to isolate spectrometer counts coming from OH signals (Fig. S17b). Ferrocene has an additional peak near 3100 cm^{-1} that comes from ferrocene alone, so this peak is not included in our analysis. After background subtraction, the integrated counts over the OH bands in CB[7] samples versus Fc@CB[7] samples is compared (Fig. S17c). Overall that the OH band counts of Fc@CB[7] show lower intensity than CB[7], which agrees with the interpretation of Fc expelling cavity water from inside CB[7].

Finally, within the Fc@CB[7] measurements, we correlate the CB_{450} and CB_{830} peak positions (lower frequencies should mean higher proportion of CB[7] cavities filled with Fc) with OH band counts (lower counts should mean higher proportion of CB[7] cavities filled with Fc). We observe a positive correlation between both CB_{450} and CB_{830} centre frequencies with OH band counts (Fig. S17d,e), which supports the hypothesis that Fc inclusion reduces OH band counts. Moreover, the CB_{450} and CB_{830} peaks centres are strongly correlated to each other (Fig. S17f), agreeing with the interpretation that different points on the solid have different degrees of Fc inclusion.

Supplementary Note 6: Ion-capping experiments with CB[5] and $FeCl_3$

To test whether we could directly modify the chemical environment of water clusters encapsulated in CB[5], we mixed CB[5] solutions with $FeCl_3$ solutions in a 1:2 molar ratio. Our aim was to cap both electronegative carbonyl portals on CB[5] with Fe^{3+} ion. With this solution we repeated the same experiment as described in Methods in the main text by placing the solution inside a flow cell and taking spectra as it dries in a nitrogen environment.

For CB[5] alone, we could measure solidified CB[5] directly on a gold substrate (Fig. S18**,** pink). However, the CB[5]-$FeCl_3$ mixture etched the gold substrate, so the sample (Fig. S18**,** green) was measured on a glass substrate that produces higher background signals (Fig. S18**,** grey).

In both H_2O and D_2O experiments with the CB[5]-$FeCl_3$ mixture, the dried samples exhibit two prominent peaks near the strong CB[*n*] vibrations around 450 cm^{-1} and 830 cm^{-1} (Fig. S18a,d). However, many narrow Raman sub-bands that were observed with CB[5] alone in H_2O and D_2O disappear in the CB[5]-$FeCl_3$ mixture. The Fe^{3+} ion thus clearly modifies the macroscopic structure of the CB[5] solid, which is expected because it interacts with the carbonyl portals, which dominate bonding processes in CB[*n*] solids (29). The disappearance of Raman bands in the CB[5]-$FeCl_3$ sample could indicate higher inter-CB[*n*] vibrational coupling within the solid, broadening previously narrow bands. We only present preliminary results here as further study is necessary to elucidate the details of this more complex structure.

The CB[5]-$FeCl_3$ sample produces narrow peaks that come from confined water. In the D_2O experiment, the CB[5]-$FeCl_3$ sample exhibits narrow OD stretch peaks (Fig. S18b) and weaker narrow OH stretch peaks (Fig. S18c**)**. The OD/OH peaks in the CB[5]-$FeCl_3$ sample are similar to the OD/OH peaks seen in CB[5] alone. For both CB[5]-$FeCl_3$ and CB[5] alone D_2O experiments, the OD peaks are stronger and greater in number than the OH peaks. However, while CB[5] alone in D_2O produced 3-4 discernible OD peaks, only 2 OD peaks are observed in the CB[5]-$FeCl_3$ sample.

Both the CB[5] alone and CB[5]-$FeCl_3$ H_2O experiments show no OD peaks (Fig. S18e) and narrow OD peaks (Fig. S18f). Just as in the D_2O experiment, the CB[5]-$FeCl_3$ H_2O experiment exhibits fewer narrow water peaks than the CB[5] sample alone H_2O experiment. While the CB[5] alone H_2O experiment shows three clear peaks (Fig. 4c), the CB[5]-$FeCl_3$ H_2O experiment only shows two clear peaks.

In summary, the CB[5]-$FeCl_3$ solids exhibit similar CB[*n*] vibrations and clustered water peaks as solids from CB[5] alone. However, the clustered water peaks reveal significant changes to the confined water chemical environment. In both the H_2O and D_2O experiments, the narrow clustered water peaks shift to lower frequencies by 200-300 cm^{-1} with the addition of $FeCl_3$, indicating stronger hydrogen bonds on water molecules. Moreover, the confined water peaks become enhanced by 5-10x with the addition of $FeCl_3$, showing enhanced Raman polarizabilities. Finally, we observe fewer confined water peaks in samples with $FeCl_3$, indicating that it could be more favourable for a water monomer to reside inside CB[5] than a water dimer.

Supplementary Figures

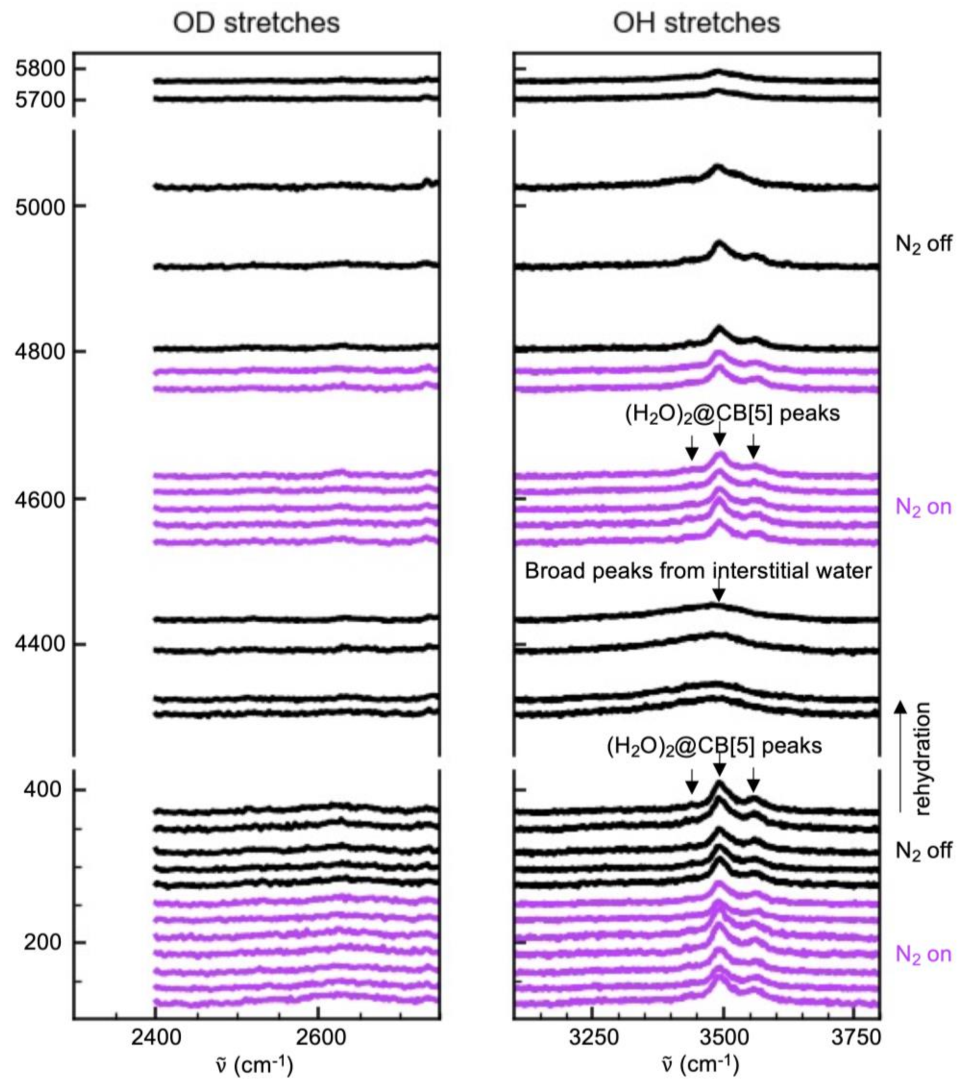

Fig. S1. Influence of nitrogen atmosphere on solid-state CB[5]. Exposing a CB[5] sample to air (black curves) causes the structured peaks observed in a nitrogen atmosphere (purple peaks) to broaden significantly as the crystal pulls water from the atmosphere inside solid interstitial regions.

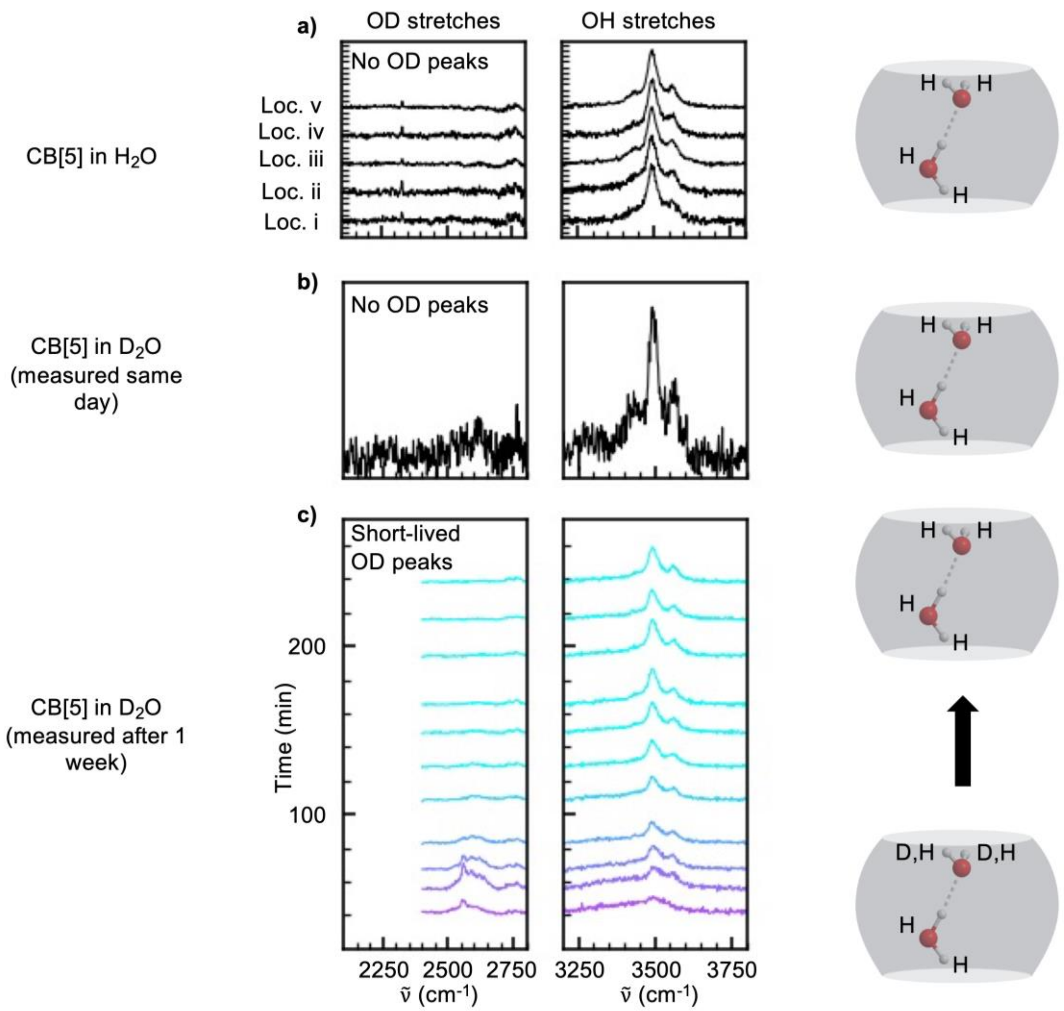

Fig. S2. Slow incorporation of deuterium into CB[5]. When CB[5] is mixed in a D_2O solution and measured on the same day, the vibrational signals match that of CB[5] in H_2O. When the same experiment is conducted after 1 week of incubation time, some OD signals are observed, but they represent only the top hydrogens switching to deuterium.

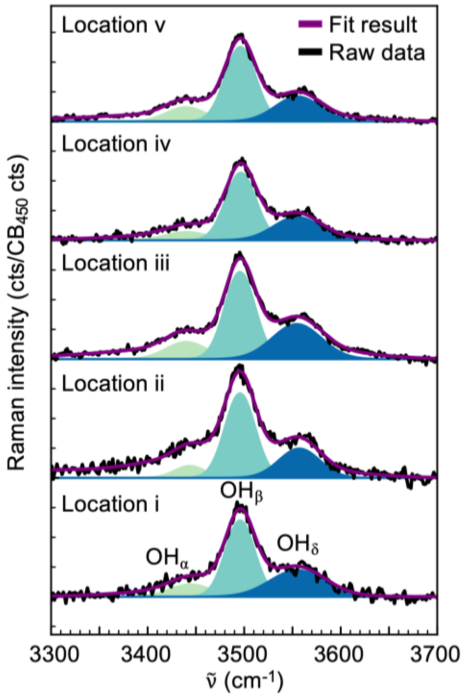

Fig. S3. Fitted OH peaks for different locations in the CB[5] H_2O experiment. The time-averaged OH spectra of five different sample locations tracked over time in the CB[5] H_2O experiment (Fig. 2a, main text) are shown alongside fitted three Gaussian peaks and fit result.

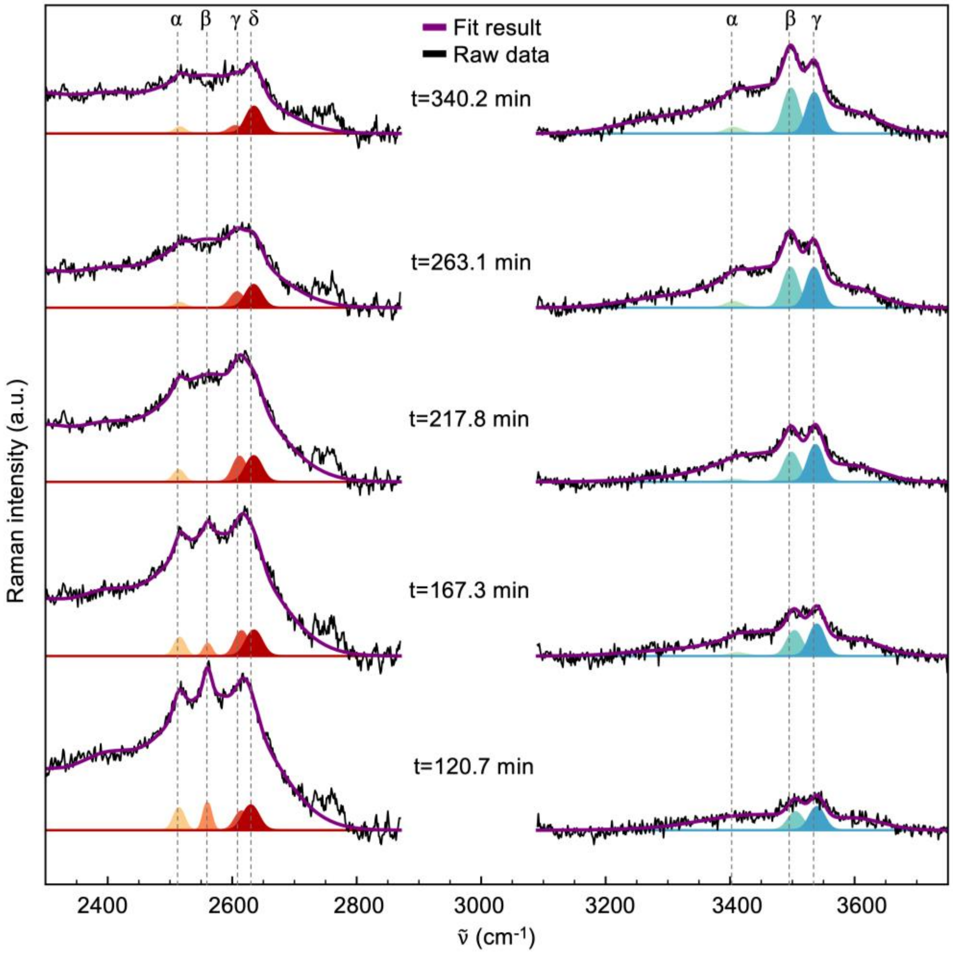

Fig. S4. Fitted OD/OH peaks in the CB[5] D_2O experiment. OD/OH spectra for the five different timepoints used in Fig. 3c of the main text are shown along with curve fits and fitted Gaussian peaks. Fitted broad bulk Gaussian peaks, representing bulk water background are omitted here for clarity.

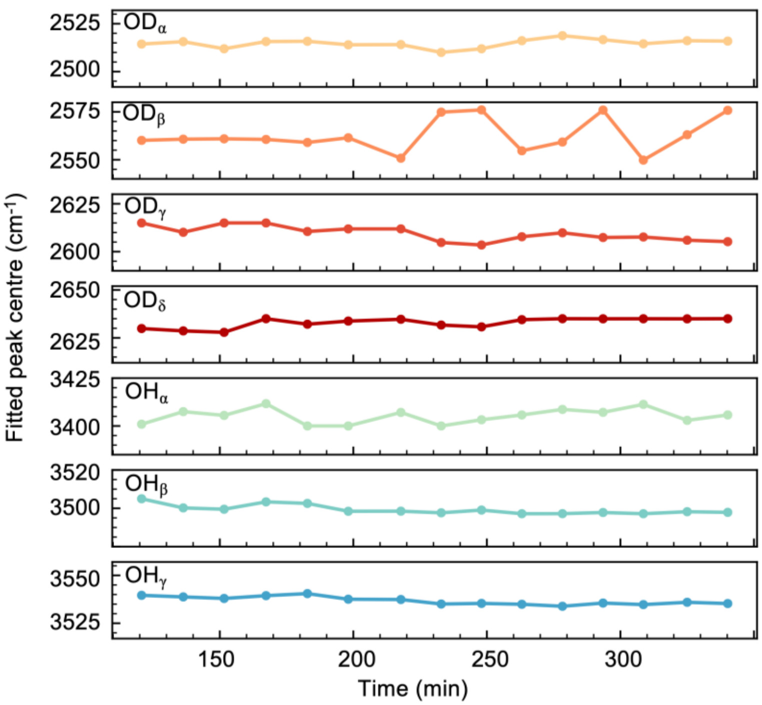

Fig. S5. CB[5] D_2O experiment fitted peak centres over time. Fitted peak centres correspond to fits from Fig. 3c of the main text and Fig. S4.

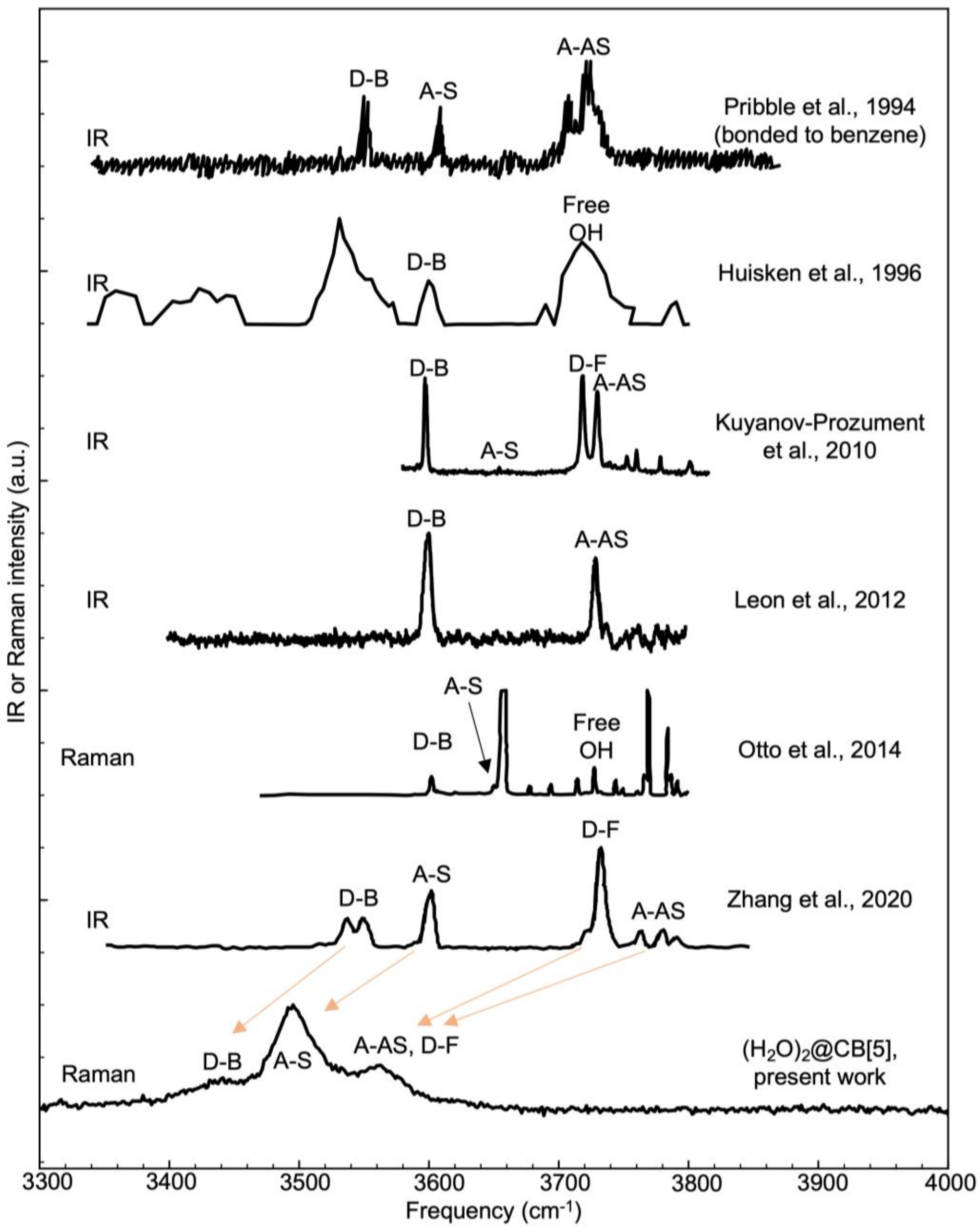

Fig. S6. Observed $(H_2O)_2$@CB[5] OH spectra compared to water dimer molecular beam bands from literature. Band assignments are written in terms of commonly-used notation in water dimer literature: D-B = donor water bound OH stretch, D-F = donor water free OH stretch, A-S = acceptor water symmetric stretch, A-AS = acceptor water asymmetric stretch. Spectra from molecular beam literature are taken from Pribble et al. (10), Huisken et al. (11), Kuyanov-Prozument et al. (12), Leon et al. (13), Otto et al. (14), and Zhang et al. (15).

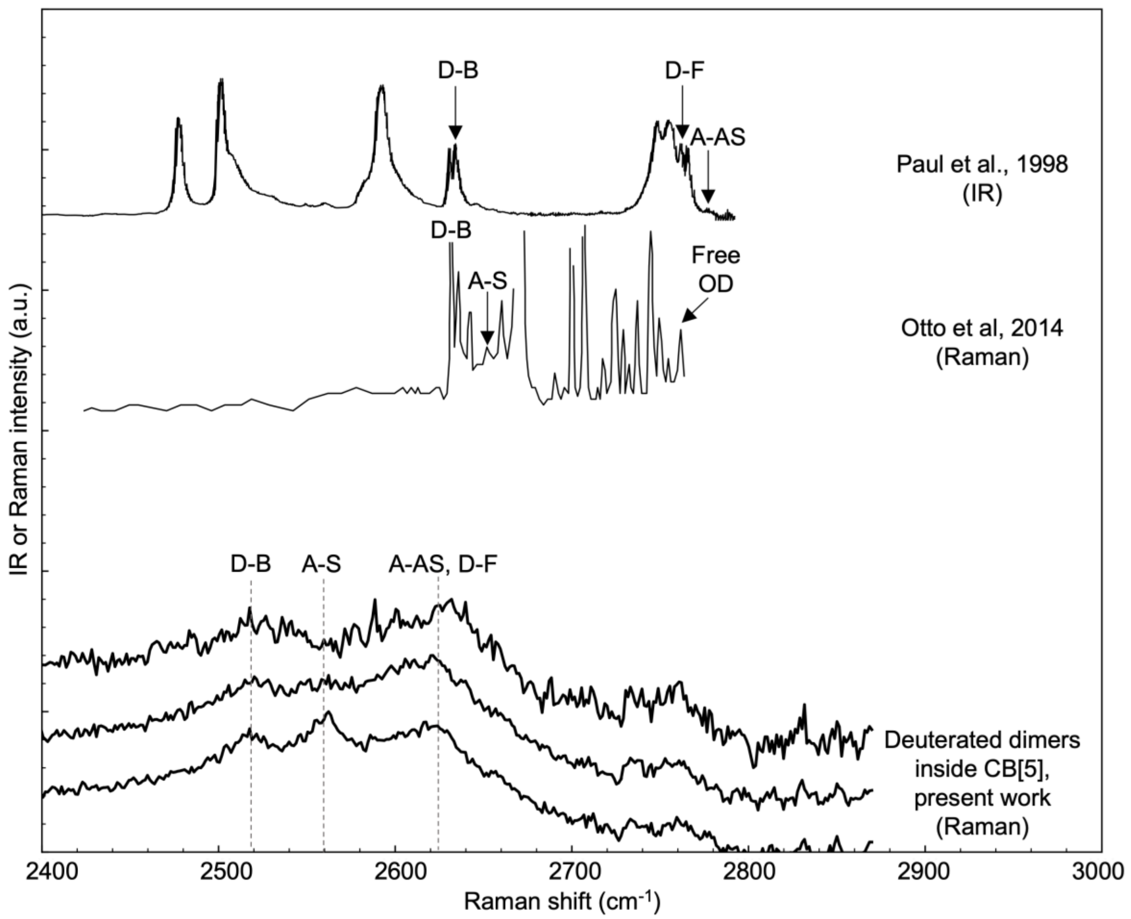

Fig. S7. Observed $(D_2O)_2$@CB[5] vibrational frequencies from CB[5] deuterated dimer experiment compared to $(D_2O)_2$ molecular beam bands from literature. As in Fig. S6, dimer bands are labeled as D-B = donor water bound OH stretch, D-F = donor water free OH stretch, A-S = acceptor water symmetric stretch, A-AS = acceptor water asymmetric stretch.

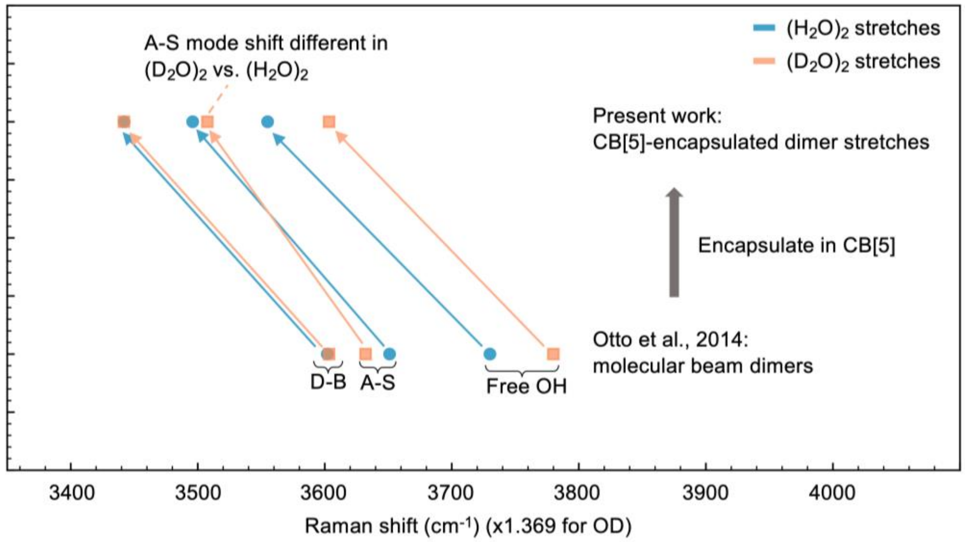

Fig. S8. Frequency shifts of $(H_2O)_2$ and $(D_2O)_2$ upon CB[5] encapsulation. The D-B, A-S, and Free OH stretches for $(H_2O)_2$ and $(D_2O)_2$ dimers are plotted for free water dimers from the study by Otto et al. (14) and for CB[5]-confined dimers from the present study. For OD and OH to be plotted on the same axis, OD stretch frequencies are multiplied by 1.369, the ratio of H_2O:D_2O monomer stretch frequencies from Otto et al. (14). After the frequency rescaling, D-B and Free OH modes shift almost identically upon CB[5] encapsulation for both $(H_2O)_2$ and $(D_2O)_2$. However, the A-S mode has a smaller redshift upon encapsulation for the deuterated dimer than expected from just a frequency axis rescaling.

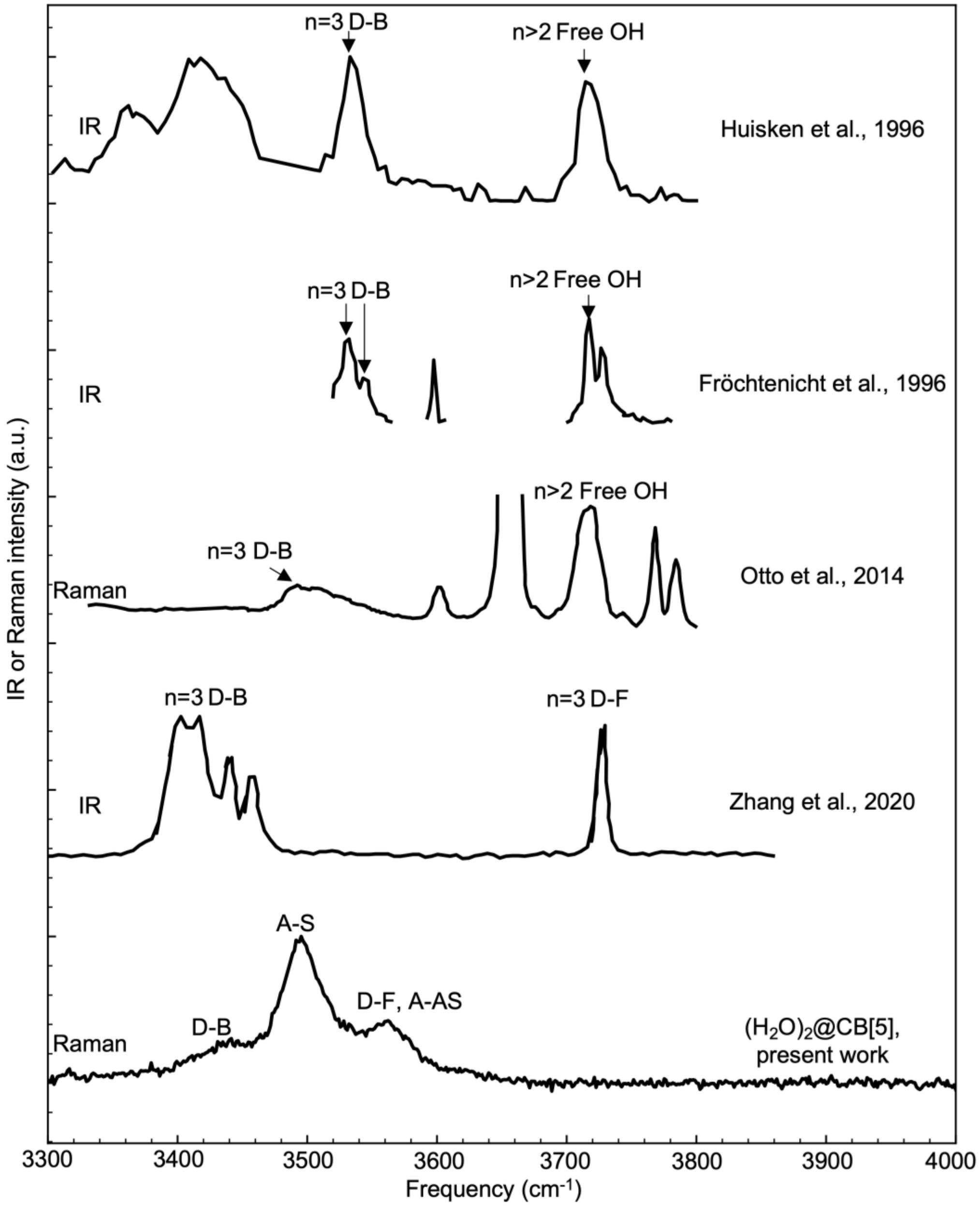

Fig. S9. Observed $(H_2O)_2$@CB[5] OH spectra compared to cyclic water trimer molecular beam bands from literature. As in Supp. Fig. 6, dimer bands are labeled as D-B = donor water bound OH stretch, D-F = donor water free OH stretch, A-S = acceptor water symmetric stretch, A-AS = acceptor water asymmetric stretch. Bands from water trimer literature are also labeled with D-B and D-F corresponding to bound and free stretches of the donor molecule, and with the cluster size *n* corresponding to the cluster $(H_2O)_n$. Spectra from literature taken from Huisken et al. (11), Fröchtenicht et al. (18), Otto et al. (14), and Zhang et al. (19).

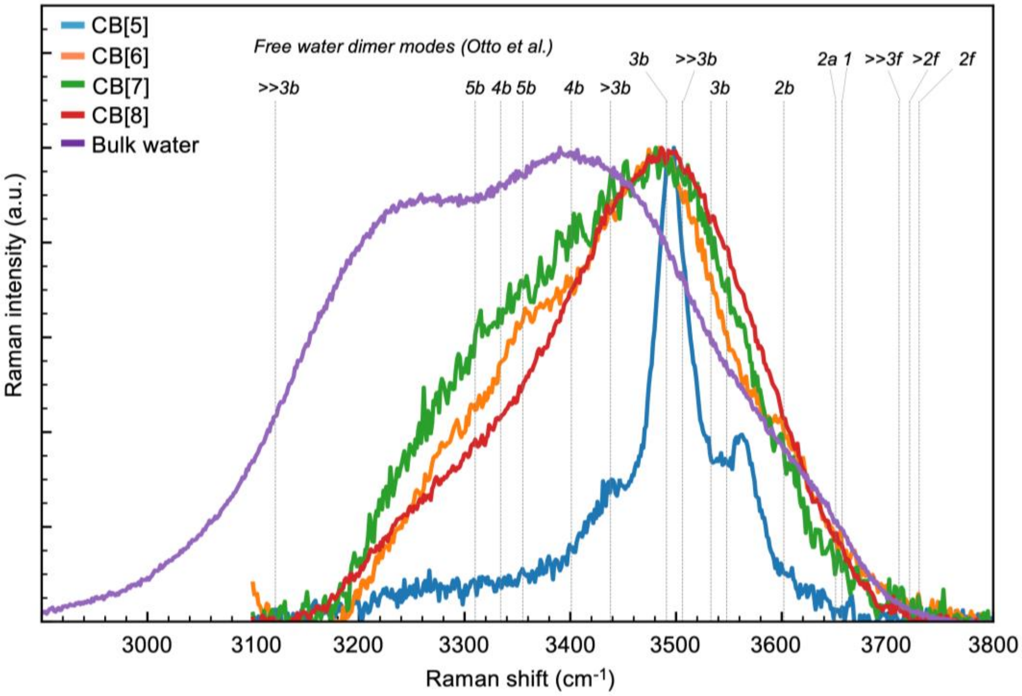

Fig. S10. CB-confined water cluster OH spectra for different CB size along with Raman spectrum of bulk H2O and free water dimer modes from Otto et al. (14)**.** Lines from Otto et al*.* are labelled consistent with the original publication; the first part of the label represents the number of water molecules in the cluster and the second part represents a free or bonded vibration.

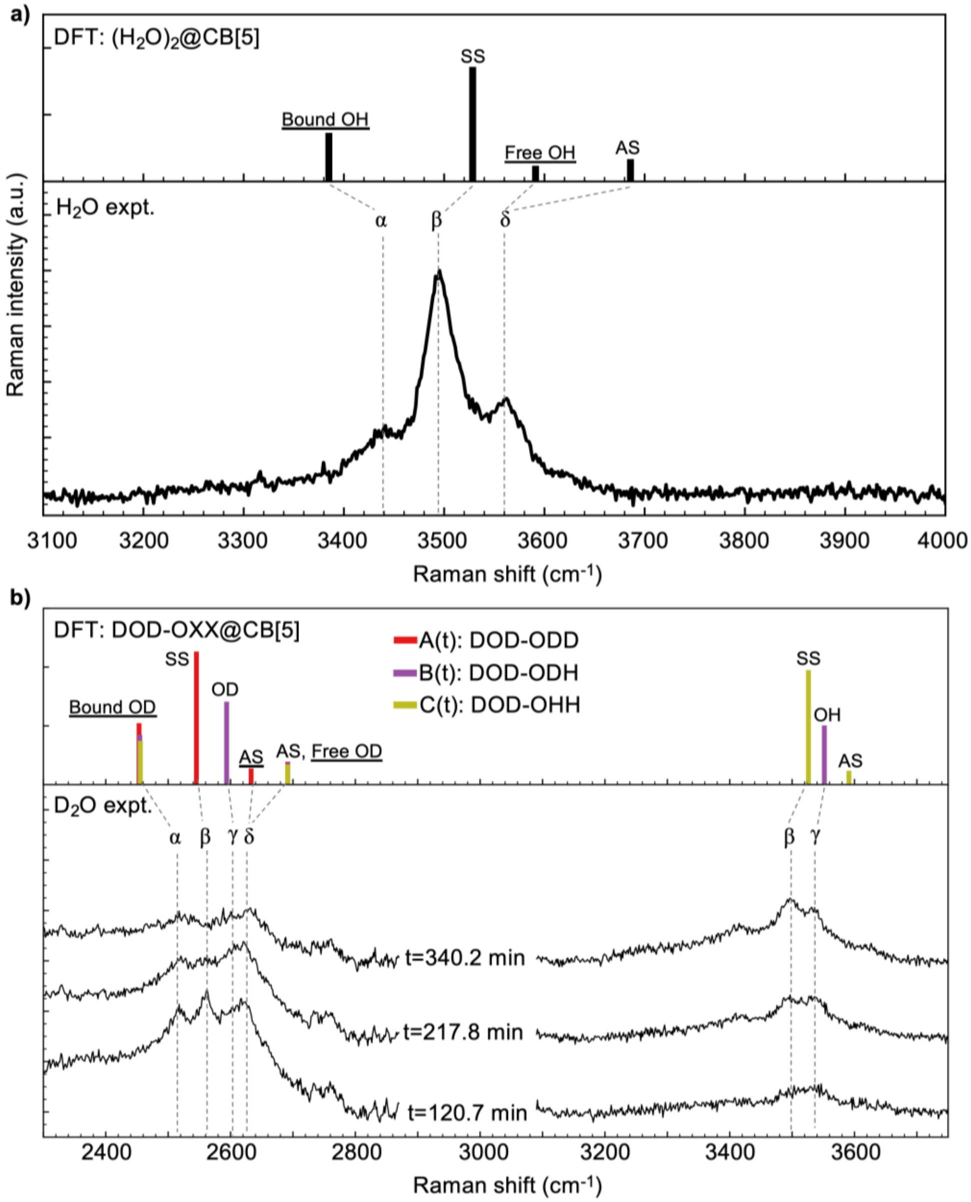

Fig. S11. Experimental spectra from H_2O and D_2O experiments compared to DFT. (a) CB[5]-confined H_2O dimer OH spectrum plotted alongside vibrational bands from DFT. (b) Experimental spectra from CB[5] D_2O experiment plotted alongside vibrational bands from isotopologues DOD-ODD, DOD-ODH, and DOD-OHH. DFT calculation used a B3LYP/6-31+G(d,p) basis set with a global scale factor of 0.952.

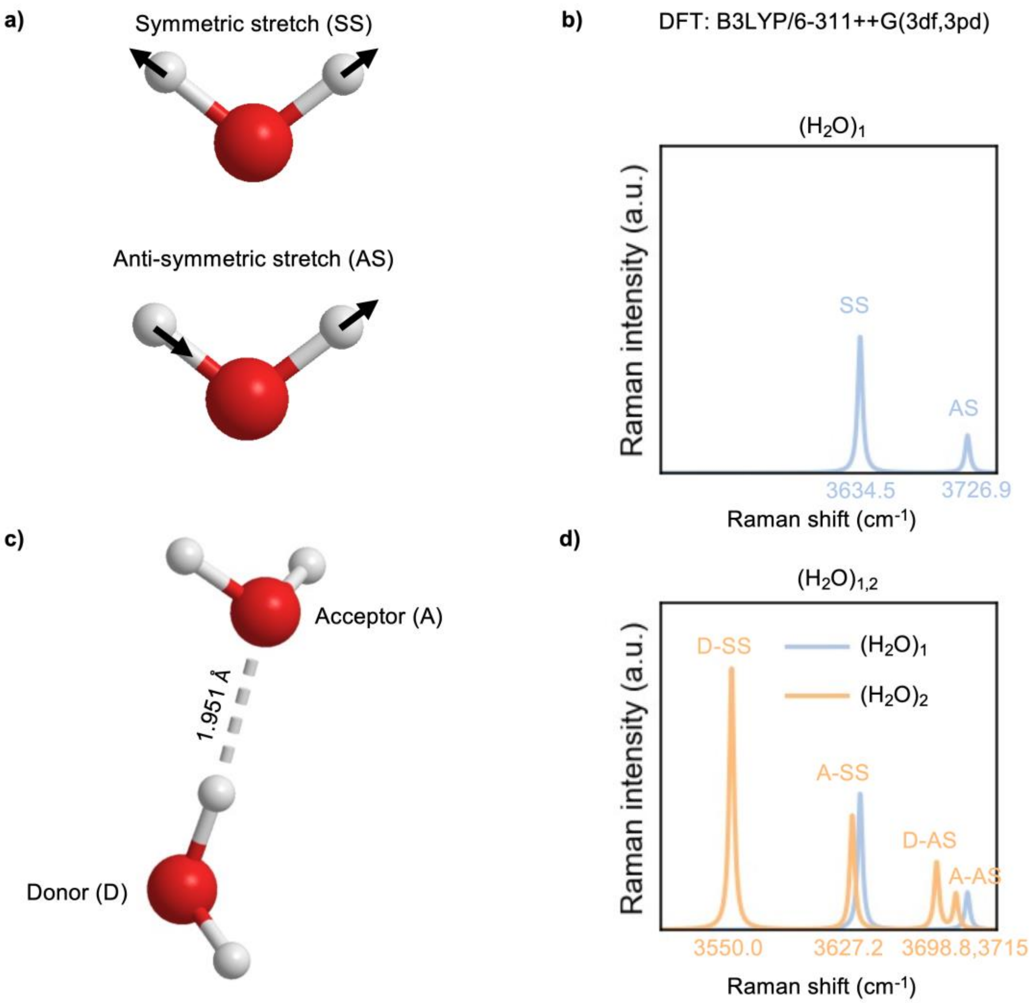

Fig. S12. OH stretches of free water dimers. (a) Two fundamental stretching vibrations of the water monomer. (b) DFT-simulated Raman spectra of free water monomer. (c) DFT-optimized geometry of a free linear water dimer. (d) DFT-simulated Raman spectra of a free water dimer. All labelled frequencies are taken from previous measurements on cold water clusters (21). All calculations done to B3LYP/6-311++G(3df,3pd) level of theory.

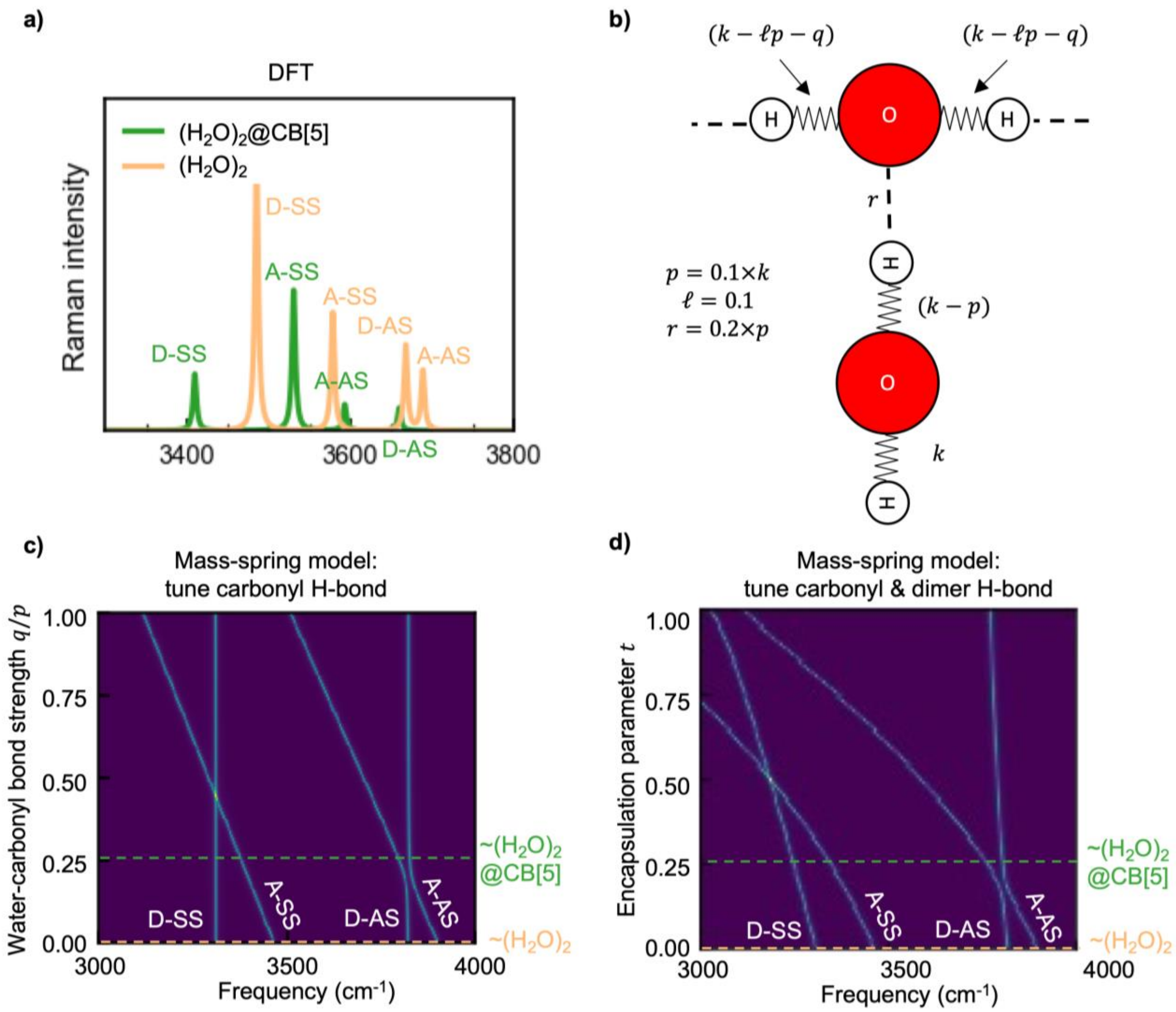

Fig. S13. Simulating water dimer@CB[5] with DFT and a mass-spring model. (a) DFT eigenfrequency comparison for free $(H_2O)_2$ *vs* $(H_2O)_2$@CB[5] (for the optimized geometry shown in Fig. 3a of the main text). (b) Schematic mass-spring model used to understand frequency shifts/ordering in (a). The model is parametrized by the unperturbed OH bond spring constant k; p and ℓp, the perturbation in spring constant for donor and acceptor, respectively from H-bond forming between them; q, the perturbation in spring constant from acceptor bonding to carbonyl; and r, the weak spring constant coupling the two molecules. (c) Mass-spring system eigenfrequencies as a function of water-carbonyl bond strength q/p. (d) Mass-spring system eigenfrequencies as both water carbonyl H-bond and dimer H-bond are increased together, parametrized by the encapsulation parameter t.

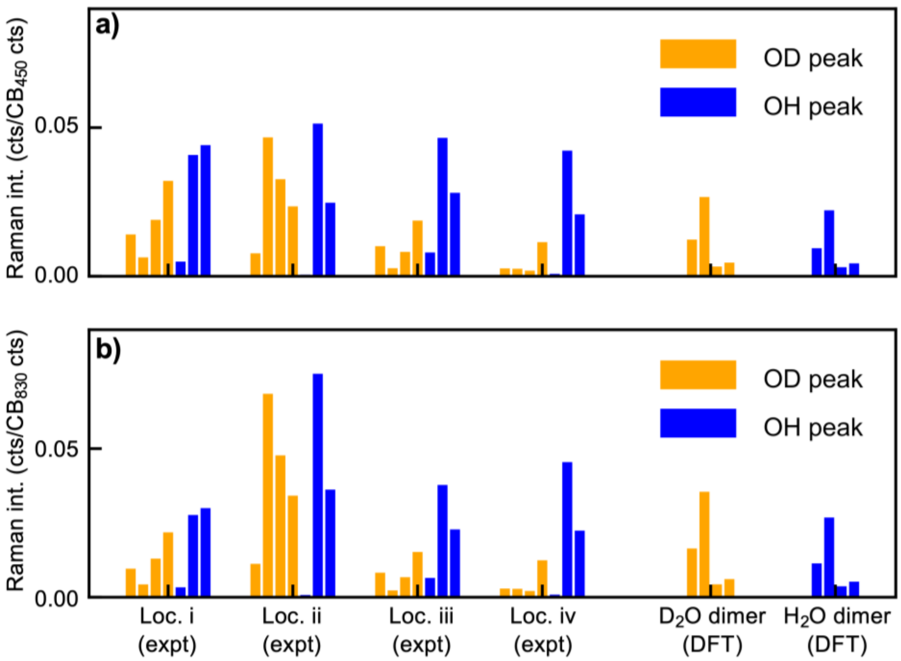

Fig. S14. DFT predicts intensity ratios between water and CB[*n*] vibrations. Left: Averaged peak intensities of OD_α, OD_β, OD_γ, OD_δ, OH_α, OH_β, and OH_γ in the D_2O experiment divided by (a) CB_{450} and (b) CB_{830} peak heights. Right: DFT-calculated intensity ratios between D_2O and H_2O dimer modes and CB_{450} and CB_{830} vibrations.

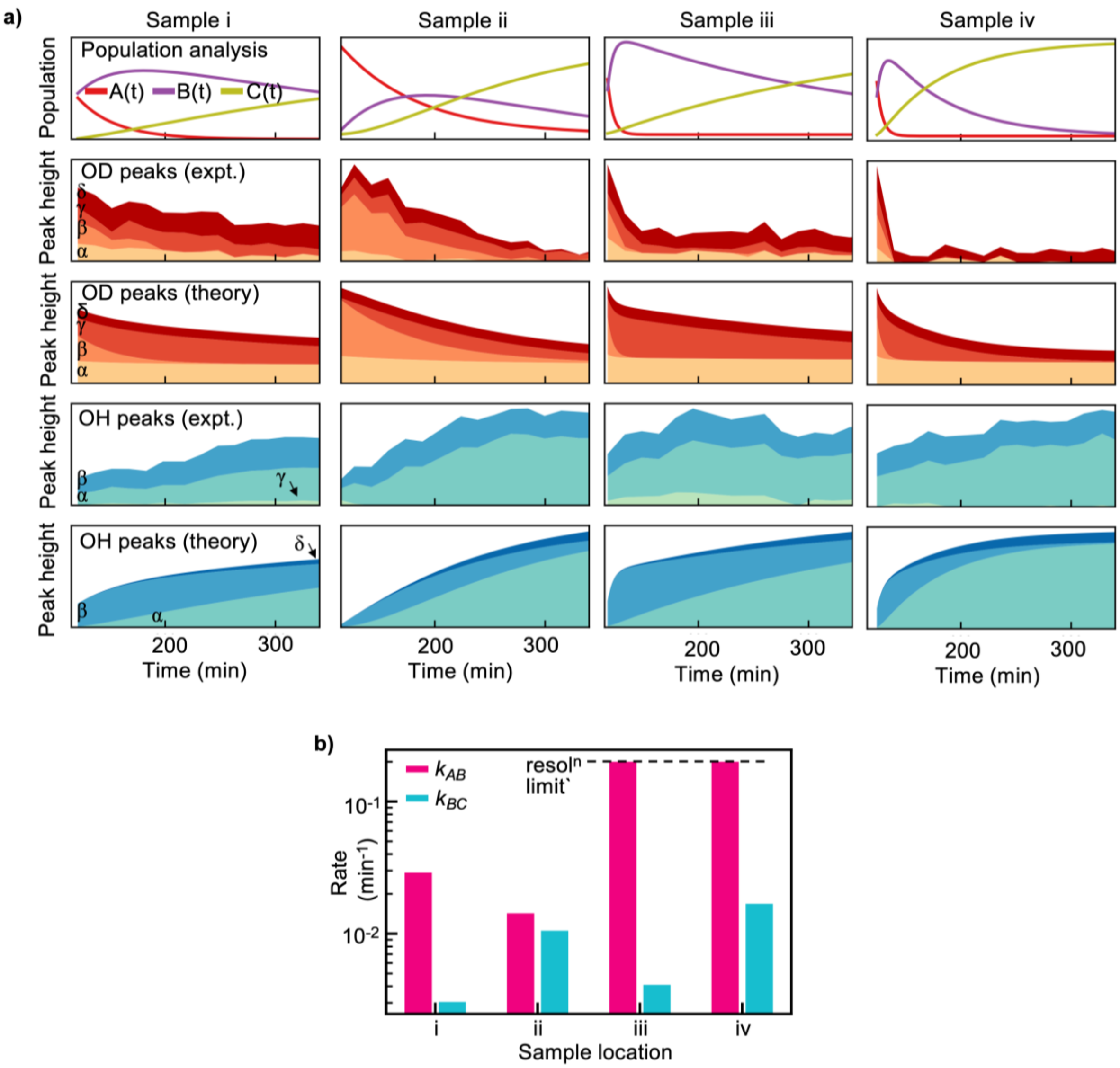

Fig. S15: Kinetic modeling over all tracked sample locations in CB[5] D_2O isotopic exchange experiment. (a) Observed OD/OH peak heights and corresponding predicted peak heights from fitting experimental data to the kinetic model described in the main text. Sample labels i-iv correspond to those used in Fig. 3e of the main text. **(b)** Fitted k_{AB} and k_{BC} indicated in (b), for four different locations on the sample (labelled i-iv).

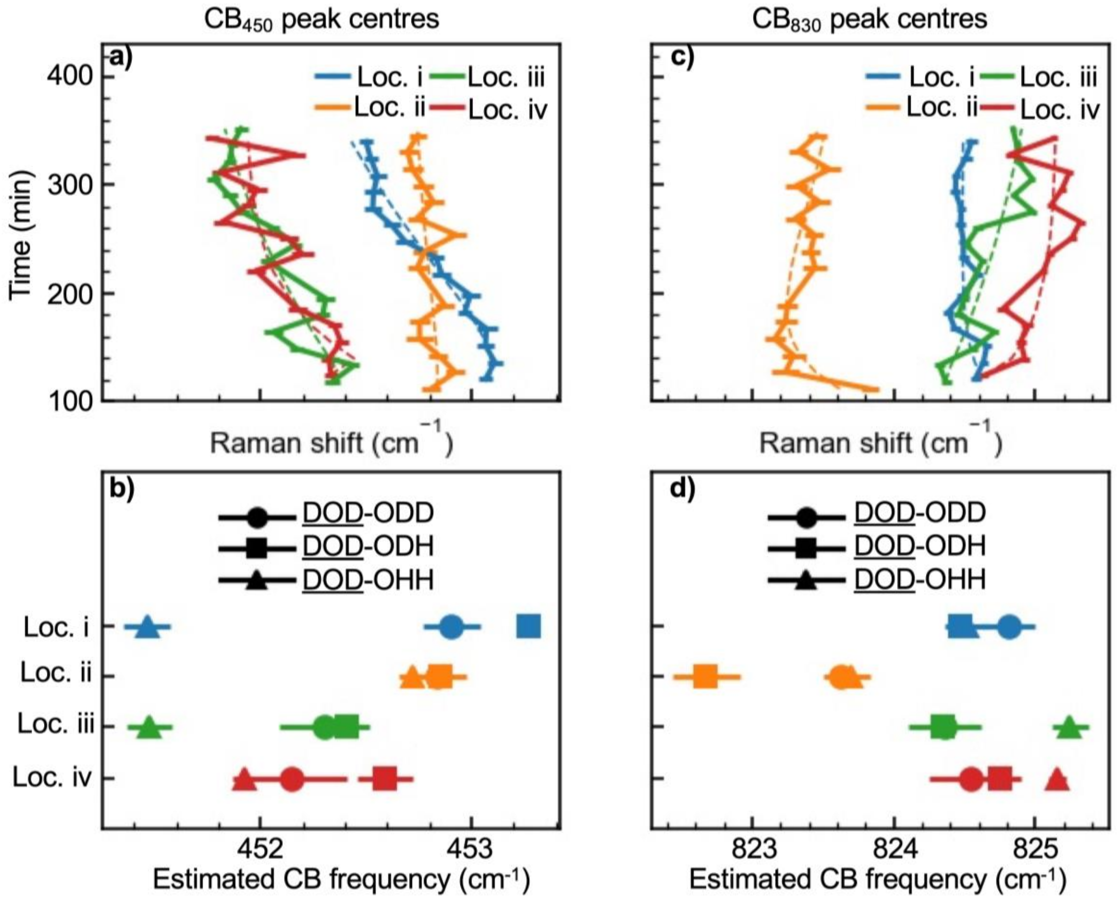

Fig. S16. Predicting CB[5] peak shifts with kinetic model results. (a,c) CB_{450} (CB_{830}) peak shifts as a function of time for four different locations on the sample. Dashed curves are generated using results from the kinetic model. (b,d) Using the kinetic model results in three different estimated CB_{450} (CB_{830}) peak frequencies for the three different isotopologues.

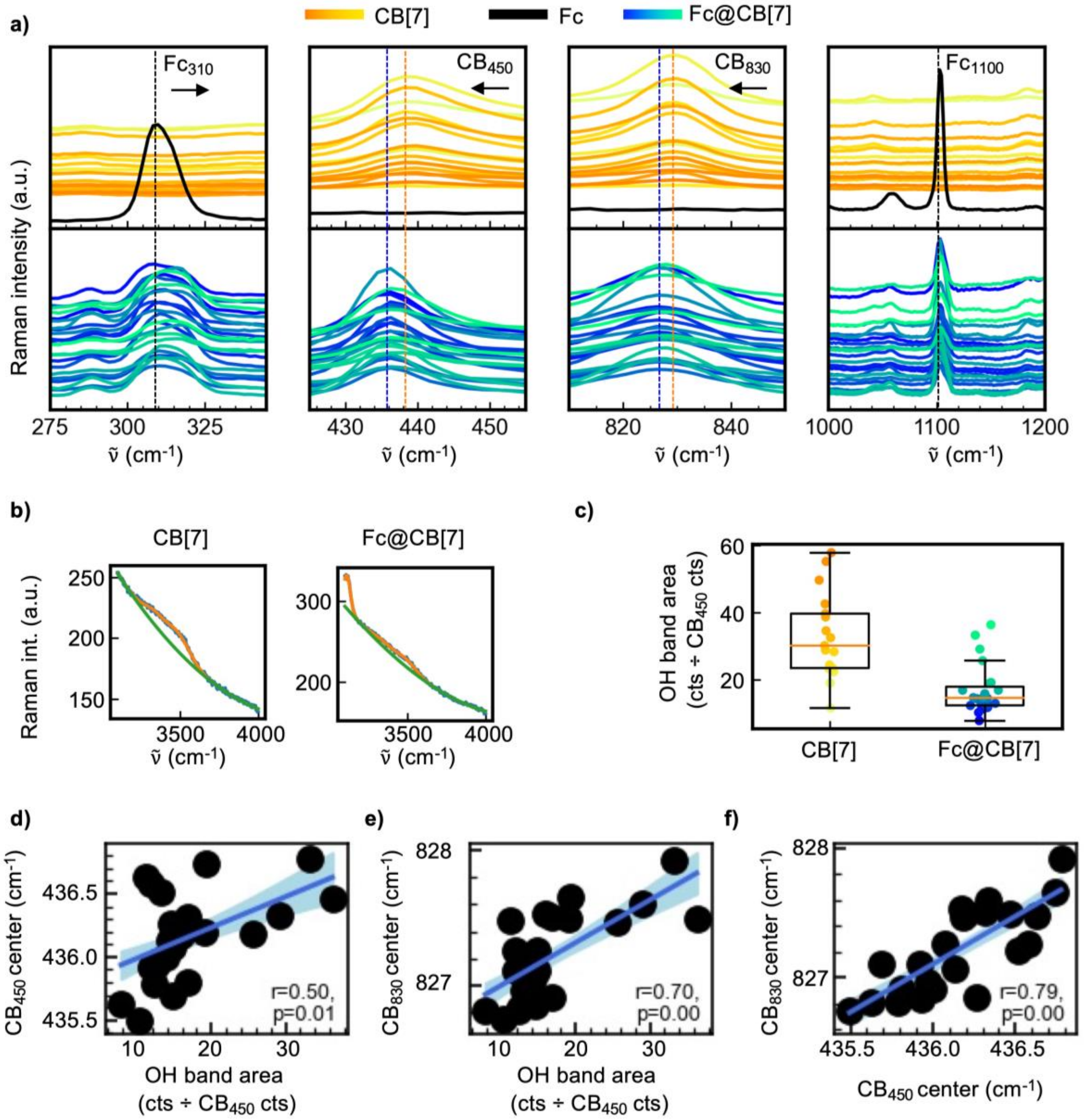

Fig. S17. Ferrocene expels cavity water in CB[7]. (a) Comparisons of Raman spectra of ferrocene (powder), flow-cell dried CB[7], and flow-cell dried Fc@CB[7]. Different spectral traces within the same colour scheme represent different locations on the same sample. (b) Representative curve fits for CB[7] and Fc@CB[7] samples. (c) Box plots showing higher average OH band area for CB[7] than for Fc@CB[7]. Each point represents a different location on the respective sample. (d,e,f) Positive correlations between CB_{450} centre and OH band area; CB_{830} centre and OH band area; and CB_{830} centre and CB_{450} centre.

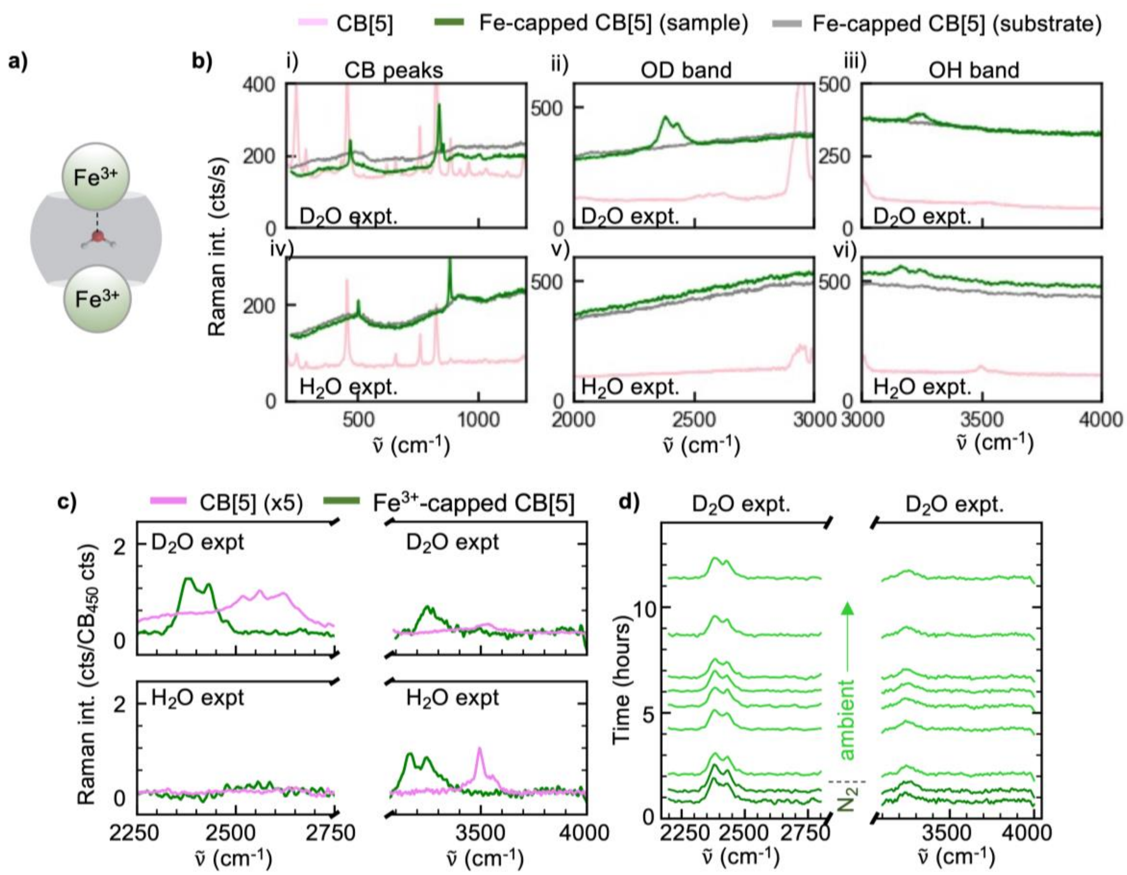

Fig. S18. Raw Raman spectral data for $FeCl_3$ experiment. (a) Schematic of potential water interactions with Fe^{3+} ions at CB[5]. **(b)** (i,iv) CB[*n*] peaks, (ii,v) OD stretch band, and (iii,vi) OH stretch bands for CB[5] samples, Fe^{3+}-capped CB[5] samples, and the substrate of the Fe^{3+}-capped CB[5] samples prepared in either D_2O (i-iii) or H_2O (iv-vi). **(c)** Raman spectra of Fe^{3+}-capped CB[5] showing strong OD/OH band peak enhancements and frequency shifts. **(d)** OD stretches from water in Fe^{3+}-capped CB[5] system show stability over 10 hours in ambient conditions.

Table S1. Information about water clusters@CB[*n*] from previous experiments and theory.

	CB[5]	CB[6]	CB[7]	CB[8]	CB[9]	CB[10]	Bulk water
**Number of waters (expt)**							
XRD (1)	1-2	3-4		11-12			
XRD (2)	2			8			
**Number of waters (theory)**							
PC analysis (3)	2	4	8	12	16	22	
MD simulation (3)	2	4	7	10	14	20	
MD simulation (4)	2.0	3.3	7.9	13.1			
MD simulation (1)	2	4	7	10			
MD simulation (5)		4					
QC calc (1)	2	4	8-9	10-12			
QC calc (6)			8				
**Hydrogen bond count** (4)	0.99	1.31	2.01	2.55			2.54
**E_{pot} (kJ/mol)** (4)	63.2	64.4	74.4	81.1			79.0

Table S2. Fitted Gaussian peak centres and FWHMs for $(H_2O)_2$@CB[5] OH spectra. Reported values and uncertainties are given by the mean and standard deviation of fitted values over five different sample locations in the CB[5] H_2O experiment.

**Peak**	**Physical interpretation**	**Peak centre (cm^{-1})**	**FWHM (cm^{-1})**
OH_α	Cavity water bound OH stretch	3441.6 ± 2.0	46.4 ± 5.0
OH_β	Portal water symmetric stretch	3495.9 ± 0.3	36.6 ± 0.6
OH_δ	Cavity water free OH stretch, portal water asymmetric stretch	3555.3 ± 0.9	57.3 ± 5.6

Table S3. Fitted Gaussian peak centres and FWHMs for the D_2O experiment. Reported values and uncertainties are given by the mean and standard deviation of fitted values over the 'cavity-confined' period from the traces in Fig. 2f (see Supp. Note 1.C.ii for fitting details). Tabulated FWHMs were those fixed in the curve-fitting process.

**Peak**	**Physical interpretation**	**Peak centre (cm^{-1})**	**FWHM (cm^{-1})**
OD_α	Cavity water bound OD stretch	2514.8 ± 2.1	20
OD_β	Portal DOD symmetric stretch	2562.9 ± 8.5	15
OD_γ	Portal OHD OD stretch	2609.4 ± 3.7	25
OD_δ	Portal&cavity DOD asymmetric stretches, cavity water bound OD stretch, portal water OD stretch	2632.9 ± 2.5	30
OH_α	Cavity water bound OH stretch	3405.2 ± 3.8	30
OH_β	Portal HOH symmetric stretch	3499.3 ± 2.4	28.26
OH_γ	Portal OHD OH stretch	3536.8 ± 2.0	28.26
OH_δ	Portal&cavity HOH asymmetric stretches, cavity water bound OH stretch, portal water OH stretch	Not observed.	Not observed.

Table S4. Comparison of $(H_2O)_2$ mode frequencies from previous molecular beam experiments with CB-confined dimer.

**Mode frequency:**	f_{D-B} **(cm^{-1})**	f_{A-S} **(cm^{-1})**	f_{D-F} **(cm^{-1})**	f_{A-AS} **(cm^{-1})**	$f_{Free\ OH}$ **(cm^{-1})**
Pribble et al., 1994 (10)	3550	3608	3708	3722	
Huisken et al., 1996 (11)	3601				3735
Kuyanov-Prozument et al., 2010 (12)	3597.4	3654.2	3729-3730	3739-3759	
Leon et al., 2012 (13)	3601.0	3670		3730-3732	
Otto et al., 2014 (14)	3602	3651			3730
Zhang et al., 2020 (15)	3537-3549	3603	3732	3764-3792	
$(H_2O)_2$@CB[5], present work	3442	3496	3555		

Table S5. Frequency shift of observed CB-confined dimer frequencies relative to previous free water $(H_2O)_2$ experiments. Frequency shift ranges are given as parenthetical ranges when the past experimental reference reports a range of values, or in the fourth column where the present work highest-frequency peak cannot distinguish between D-F and A-AS vibrations.

**Mode frequency shift:**	$f_{D-B}^{CB[5]} - f_{D-B}^{free}$ **(cm^{-1})**	$f_{A-S}^{CB[5]} - f_{A-S}^{free}$ **(cm^{-1})**	$f_{D-F;A-AS}^{CB[5]} - f_{D-F;A-AS;Free\ OH}^{free}$ **(cm^{-1})**
Pribble et al., 1994 (10)	-108	-112	(-153,-167)
Huisken et al., 1996 (11)	-159		-180
Kuyanov-Prozument et al., 2010 (12)	-155.4	-158.2	(-174,-204)
Leon et al., 2012 (13)	-159	-174	(-175,-177)
Otto et al., 2014 (14)	-160	-155	-175
Zhang et al., 2020 (15)	(-95,-107)	-107	(-177,-237)

Table S6. Comparison of molecular beam $(D_2O)_2$ mode frequencies from previous experiments with CB-confined dimer. Note that the reported $(D_2O)_2$@CB[5] frequencies have rather large uncertainties (Table S3) due to the presence of multiple isotopologues.

<table>
<tr><th>Mode frequency:</th><th>f_{D-B} (cm^{-1})</th><th>f_{A-S} (cm^{-1})</th><th>f_{D-F} (cm^{-1})</th><th>f_{A-AS} (cm^{-1})</th><th>$f_{Free\ OD}$ (cm^{-1})</th></tr>
<tr><td>Paul et al., 1998 (17)</td><td>2632</td><td></td><td>2765</td><td>2783</td><td></td></tr>
<tr><td>Otto et al., 2014 (14)</td><td>2633</td><td>2654</td><td></td><td></td><td>2762</td></tr>
<tr><td>$(D_2O)_2$@CB[5], present work</td><td>2515</td><td>2563</td><td colspan="3">2633</td></tr>
</table>

Table S7. Frequency shift of observed CB-confined D_2O dimer frequencies relative to previous molecular beam $(D_2O)_2$ experiments. Frequency shift ranges are given as parenthetical ranges in the fourth column where the present work's highest-frequency peak cannot distinguish between D-F and A-AS vibrations.

**Mode frequency shift:**	$f_{D-B}^{CB[5]} - f_{D-B}^{free}$ **(cm^{-1})**	$f_{A-S}^{CB[5]} - f_{A-S}^{free}$ **(cm^{-1})**	$f_{D-F;A-AS}^{CB[5]} - f_{D-F;A-AS;Free\ OD}^{free}$ **(cm^{-1})**
Paul et al., 1998 (17)	-117		(-132,-150)
Otto et al., 2014 (14)	-118	-91	-129

Table S8: Bond lengths computed in $(H_2O)_2$@CB[5] for present work vs. Biedermann et al. (4) and in Grishaeva et al. (1)

Bond length	Present work	Biedermann et al. (4)	Grishaeva et al. (1)
Portal water – carbonyl H-bond (Å)	2.016, 2.018	2.185, 2.102	1.98
Portal water – carbonyl O-O distance (Å)	2.920, 2.926	3.00	Not reported
Cavity water H – acceptor water O H-bond (Å)	1.824	1.853	1.77
Portal water – cavity water O-O distance (Å)	2.782	2.789	Not reported
Portal water OH bond length (Å)	0.972, 0.973	0.971, 0.972	Not reported
Cavity water bound OH bond length (Å)	0.980	0.978	Not reported
Cavity water free OH bond length (Å)	0.965	0.968	Not reported

SI References

1. T. N. Grishaeva, A. N. Masliy, A. M. Kuznetsov, Water structuring inside the cavities of cucurbit[n]urils (n=5-8): a quantum-chemical forecast. *J. Incl. Phenom. Macrocycl. Chem.* **89**, 299–313 (2017).
2. J. Kim, *et al.*, New Cucurbituril Homologues: Syntheses, Isolation, Characterization, and X-ray Crystal Structures of Cucurbit[n]uril (n=5, 7, and 8). *J. Am. Chem. Soc.* **122**, 540–541 (2000).
3. K. I. Assaf, W. M. Nau, Cucurbiturils: from synthesis to high-affinity binding and catalysis. *Chem. Soc. Rev.* **44**, 394–418 (2015).
4. F. Biedermann, V. D. Uzunova, O. A. Scherman, W. M. Nau, A. D. Simone, Release of High-Energy Water as an Essential Driving Force for the High-Affinity Binding of Cucurbit[n]urils. *J. Am. Chem. Soc.* **134**, 15318–15323 (2012).
5. K. B. Tarmyshov, F. Müller-Plathe, Ion Binding to Cucurbit[6]uril: Structure and Dynamics. *J. Phys. Chem. B* **110**, 14463–14468 (2006).
6. N. S. Venkataramanan, A. Suvitha, R. Sahara, Structure, stability, and nature of bonding between high energy water clusters confined inside cucurbituril: A computational study. *Comput. Theor. Chem.* **1148**, 44–54 (2019).
7. V. V. Bakovets, E. A. Kovalenko, T. P. Chusova, L. N. Zelenina, P. E. Plyusnin, State of water in CB[6] and CB[8] cavitands. *Russ. Chem. Bull.* **62**, 2109–2115 (2013).
8. M. Erko, G. H. Findenegg, N. Cade, A. G. Michette, O. Paris, Confinement-induced structural changes of water studied by Raman scattering. *Phys. Rev. B* **84**, 104205 (2011).
9. F. G. Alabarse, *et al.*, Freezing of Water Confined at the Nanoscale. *Phys. Rev. Lett.* **109**, 35701 (2012).
10. R. N. Pribble, T. S. Zwier, Size-Specific Infrared Spectra of Benzene-(H2O)n Clusters (n = 1 through 7): Evidence for Noncyclic (H2O)n Structures. *Science* **265**, 75–79 (1994).
11. F. Huisken, M. Kaloudis, A. Kulcke, Infrared spectroscopy of small size-selected water clusters. *J. Chem. Phys.* **104**, 17–25 (1996).
12. K. Kuyanov-Prozument, M. Y. Choi, A. F. Vilesov, Spectrum and infrared intensities of OH-stretching bands of water dimers. *J. Chem. Phys.* **132**, 014304 (2010).
13. I. León, R. Montero, F. Castaño, A. Longarte, J. A. Fernández, Mass-Resolved Infrared Spectroscopy of Complexes without Chromophore by Nonresonant Femtosecond Ionization Detection. *J. Phys. Chem. A* **116**, 6798–6803 (2012).
14. K. E. Otto, Z. Xue, P. Zielke, M. A. Suhm, The Raman spectrum of isolated water clusters. *Phys. Chem. Chem. Phys.* **16**, 9849 (2014).
15. B. Zhang, *et al.*, Infrared Spectroscopy of Neutral Water Dimer Based on a Tunable Vacuum Ultraviolet Free Electron Laser. *J. Phys. Chem. Lett.* **11**, 851–855 (2020).
16. Y. Ikemoto, *et al.*, Infrared Spectra and Hydrogen-Bond Configurations of Water Molecules at the Interface of Water-Insoluble Polymers under Humidified Conditions. *J. Phys. Chem. B* **126**, 4143–4151 (2022).
17. J. B. Paul, R. A. Provencal, C. Chapo, A. Petterson, R. J. Saykally, Infrared cavity ringdown spectroscopy of water clusters: O–D stretching bands. *J. Chem. Phys.* **109**, 10201–10206 (1998).
18. R. Fröchtenicht, M. Kaloudis, M. Koch, F. Huisken, Vibrational spectroscopy of small water complexes embedded in large liquid helium clusters. *J. Chem. Phys.* **105**, 6128–6140 (1996).
19. B. Zhang, *et al.*, Infrared spectroscopy of neutral water clusters at finite temperature: Evidence for a noncyclic pentamer. *Proc. Natl. Acad. Sci.* **117**, 15423–15428 (2020).
20. U. Buck, F. Huisken, Infrared Spectroscopy of Size-Selected Water and Methanol Clusters. *Chem. Rev.* **100**, 3863–3890 (2000).
21. L. Fredin, B. Nelander, G. Ribbegård, Infrared spectrum of the water dimer in solid nitrogen. I. Assignment and force constant calculations. *J. Chem. Phys.* **66**, 4065–4072 (1977).
22. T. R. Dyke, J. S. Muenter, Microwave spectrum and structure of hydrogen bonded water dimer. *J. Chem. Phys.* **60**, 2929–2930 (1974).

23. A. Mukhopadhyay, S. S. Xantheas, R. J. Saykally, The water dimer II: Theoretical investigations. *Chem. Phys. Lett.* **700**, 163–175 (2018).
24. W. Klopper, J. G. C. M. van Duijneveldt-van de Rijdt, F. B. van Duijneveldt, Computational determination of equilibrium geometry and dissociation energy of the water dimer. *Phys. Chem. Chem. Phys.* **2**, 2227–2234 (2000).
25. T. K. Ghanty, V. N. Staroverov, P. R. Koren, E. R. Davidson, Is the Hydrogen Bond in Water Dimer and Ice Covalent? *J. Am. Chem. Soc.* **122**, 1210–1214 (2000).
26. U. Bergmann, *et al.*, Nearest-neighbor oxygen distances in liquid water and ice observed by x-ray Raman based extended x-ray absorption fine structure. *J. Chem. Phys.* **127**, 174504 (2007).
27. A. C. Gomes, *et al.*, Solid-state study of the structure and host-guest chemistry of cucurbituril-ferrocene inclusion complexes. *Dalton Trans.* **45**, 17042–17052 (2016).
28. Y. Chen, A. Klimczak, E. Galoppini, J. V. Lockard, Structural interrogation of a cucurbit[7]uril-ferrocene host-guest complex in the solid state: a Raman spectroscopy study. *RSC Adv* **3**, 1354–1358 (2013).
29. D. Bardelang, *et al.*, Cucurbit[n]urils (n= 5-8): A Comprehensive Solid State Study. *Cryst. Growth Des.* **11**, 5598–5614 (2011).
